# Optimization and *In Vivo* Profiling of a Refined Rat Model of Walker 256 Breast Cancer Cell-Induced Bone Pain Using Behavioral, Radiological, Histological, Immunohistochemical and Pharmacological Methods

**DOI:** 10.3389/fphar.2017.00442

**Published:** 2017-07-06

**Authors:** Priyank Shenoy, Andy Kuo, Irina Vetter, Maree T. Smith

**Affiliations:** ^1^Centre for Integrated Preclinical Drug Development, Centre for Clinical Research, The University of Queensland, BrisbaneQLD, Australia; ^2^School of Biomedical Sciences, The University of Queensland, BrisbaneQLD, Australia; ^3^Institute for Molecular Bioscience, The University of Queensland, BrisbaneQLD, Australia; ^4^School of Pharmacy, The University of Queensland, BrisbaneQLD, Australia

**Keywords:** breast cancer, bony metastases, bone pain, Walker 256 cells, Wistar Han female rat model

## Abstract

In the majority of patients with advanced breast cancer, there is metastatic spread to bones resulting in pain. Clinically available drug treatments for alleviation of breast cancer-induced bone pain (BCIBP) often produce inadequate pain relief due to dose-limiting side-effects. A major impediment to the discovery of novel well-tolerated analgesic agents for the relief of pain due to bony metastases is the fact that most cancer-induced bone pain models in rodents relied on the systemic injection of cancer cells, causing widespread formation of cancer metastases and poor general animal health. Herein, we have established an optimized, clinically relevant Wistar Han female rat model of breast cancer induced bone pain which was characterized using behavioral assessments, radiology, histology, immunohistochemistry and pharmacological methods. In this model that is based on unilateral intra-tibial injection (ITI) of Walker 256 carcinoma cells, animals maintained good health for at least 66 days post-ITI. The temporal development of hindpaw hypersensitivity depended on the initial number of Walker 256 cells inoculated in the tibiae. Hindpaw hypersensitivity resolved after approximately 25 days, in the continued presence of bone tumors as evidenced by *ex vivo* histology, micro-computed tomography scans and immunohistochemical assessments of tibiae. A possible role for the endogenous opioid system as an internal factor mediating the self-resolving nature of BCIBP was identified based upon the observation that naloxone, a non-selective opioid antagonist, caused the re-emergence of hindpaw hypersensitivity. Bolus dose injections of morphine, gabapentin, amitriptyline and meloxicam all alleviated hindpaw hypersensitivity in a dose-dependent manner. This is a first systematic pharmacological profiling of this model by testing standard analgesic drugs from four important diverse classes, which are used to treat cancer induced bone pain in the clinical setting. Our refined rat model more closely mimics the pathophysiology of this condition in humans and hence is well-suited for probing the mechanisms underpinning breast cancer induced bone pain. In addition, the model may be suitable for efficacy profiling of new molecules from drug discovery programs with potential to be developed as novel agents for alleviation of intractable pain associated with disseminated breast cancer induced bony metastases.

## Introduction

Unrelenting pain in patients with advanced cancer significantly reduces quality of life (Bu et al., 2014). Metastases of cancer cells to the skeleton often causes pain in breast cancer patients (Bu et al., 2014) with over 70% of patients with terminal breast cancer having bony metastases (Coleman, 2006; Currie et al., 2013; Cleeland et al., 2016). Bony metastases are often asymptomatic initially and are usually diagnosed following the occurrence of bone pain or skeletal complications involving damage to the bone structure (Cleeland et al., 2016). In the later stages of metastatic breast cancer, bone pain can become excruciating making it very difficult to treat (Kane et al., 2015). Knowledge on the precise mechanisms by which breast and other cancers metastasize to bone and produce pain is incomplete (Zhu et al., 2015). It is somewhat counter-intuitive that primary breast tumors at the original site cause less or no pain, yet once these cells have metastasized to bone, patients may suffer excruciating pain (Lozano-Ondoua et al., 2013). In the clinical setting, non-steroidal anti-inflammatory drugs (NSAIDs), strong opioids, medications that inhibit the activity of osteoclasts like bisphosphonates, bone targeted monoclonal antibodies, radiation therapy and surgical management are the mainstay of pharmacotherapeutic treatment of BCIBP (Kane et al., 2015; Milgrom et al., 2017). A major challenge involved in understanding the interplay of mechanisms underpinning the pathobiology of BCIBP is establishment of a suitable rodent model which exhibits characteristics similar to those of BCIBP in humans (Slosky et al., 2015). Until the late 20th century, CIBP models in animals were initiated by systemically injecting the cancer cells, causing poor animal health due to tumor in the liver and lungs as well as random and multi-sited bone deposits (Urch, 2004). Subsequently, models involving local injection of breast cancer cells within a single bone have proven successful, avoiding the spread of tumors systemically to the highly perfused organs or neighboring soft tissue (Schwei et al., 1999).

The unique contribution of our study described herein is establishment of a clinically relevant, optimized Wistar Han female rat model of BCIBP that we have extensively characterized using behavioral, pharmacological, radiological (micro-computed tomography, μCT), histological and immunohistochemical methods. Our findings show for the first time, that the severity and nature of mechanical pain hypersensitivity behaviors developed in the hindpaws of female Wistar Han rats depend on the initial number of Walker 256 (W256) cells given by unilateral ITI. Additionally, we have thoroughly assessed and documented general animal health in a temporal manner in the same animals for up to 66 days post-ITI. A role for upregulated opioidergic signaling in the spontaneous resolution of pain hypersensitivities observed at later stages of the model in the continued presence of bone cancer disease was investigated for the first time in this model. To the best of our knowledge, our pharmacological data are the first ‘back translation’ profiling of this rat model of BCIBP, showing analgesic efficacy of single bolus doses of clinically available analgesic drugs (morphine, meloxicam) and adjuvant agents (gabapentin, amitriptyline) used to treat various types of chronic pain.

## Materials and Methods

### Drugs, Chemicals and Reagents

Morphine (DBL^TM^ morphine sulfate injection BP – 30 mg in 1 mL) was procured from Hospira Pty Ltd. (Melbourne, VIC, Australia). Gabapentin was kindly provided by Dr. Ben Ross, School of Pharmacy, The University of Queensland (Brisbane, QLD, Australia). Amitriptyline (amitriptyline hydrochloride), meloxicam (meloxicam sodium salt hydrate), naloxone (naloxone hydrochloride dihydrate), Triton^TM^ X-100, Tween 20, paraformaldehyde (PFA) and bovine serum albumin (BSA) were procured from Sigma–Aldrich^®^ (Castle Hill, NSW, Australia). Isoflurane (IsoFlo^TM^) was procured from Abbott Australasia Pty. Ltd. (Macquarie Park, NSW, Australia). Medical oxygen and medical carbon dioxide were procured from Coregas Pty Ltd. (Yennora, NSW, Australia). Triple antibiotic powder (Tricin^®^) was procured from Jurox Pty Ltd. (Rutherford, NSW, Australia). Benzylpenicillin (BenPen^TM^, benzylpenicillin sodium for injection) was procured from CSL Ltd. (Melbourne, VIC, Australia). Pentobarbitone (Lethabarb^®^, pentobarbitone sodium) was procured from Virbac (Australia) Pty Ltd. (Penrith, NSW, Australia). Eye ointment (Refresh Night Time^®^) was purchased from Allergan Australia Pty Ltd. (Gordon, NSW, Australia). Ethylenediaminetetraacetic acid, disodium salt, dihydrate (UltraPure^TM^ EDTA), 4′,6-diamidino-2-phenylindole, dihydrochloride (DAPI), Prolong^®^ Gold antifade reagent, phosphate-buffered saline (PBS), medium 199 (1×), horse serum, Dulbecco’s phosphate-buffered saline (DPBS, 1×) and 0.25% trypsin-EDTA (1×) were purchased from Thermo Fisher Scientific Australia Pty Ltd. (Scoresby, VIC, Australia). Normal goat serum (NGS) was purchased from Cell Signaling Technology^®^ (Danvers, MA, United States). Tissue-Tek^®^ O.C.T. Compound was procured from ProSciTech Pty Ltd. (Kirwan, QLD, Australia). Water for injection BP was purchased from Pfizer Australia Pty Ltd. (West Ryde, NSW, Australia). Ten percent neutral-buffered formalin (NBF) was purchased from Australian Chemical Reagents (Moorooka, QLD, Australia). Methanol and acetone were purchased from EMD Millipore Corporation (Billerica, MA, United States).

### Cell Culture

W256 breast cancer cells [LLC-WRC 256 (ATCC^®^ CCL-38^TM^)] at passage number 290 were procured from the American Type Culture Collection (ATCC; Manassas, VA, United States). The cells were cultured and passaged following the ATCC guidelines. Cells within passage number 292–319 were used in the present study. To summarize, the cells were thawed from the frozen stocks and cultured in 75 cm^2^ Cellstar^®^ flasks (Greiner bio-one) at 37°C (5% CO_2_: 95% atmospheric air) in 20 mL of Medium 199 (1×) supplemented with 5% horse serum. For detachment of cells, they were initially rinsed gently with 3 mL of DPBS (1×), followed by trypsinization using 2 mL of 0.25% trypsin-EDTA (1×). The cells thus detached were collected by centrifugation with 8 mL of medium for 4 min at 200 × *g*. The resultant supernatant was discarded, the pellet was re-suspended in 3 mL of DPBS and cell counting was performed using a hemocytometer. After re-centrifuging the pellet for 4 min at 200 × *g*, the cells obtained were suspended in DPBS in the required concentration (4 × 10^3^, 1.5 × 10^4^, 4 × 10^4^, 1.5 × 10^5^ and 4 × 10^5^ cells/10 μL DPBS). HK W256 cells were prepared in the same way as the live cells but with an additional step involving heating for 15 min at 90°C.

### Animals

Female Wistar Han (HsdBrlHan) rats used in these experiments were procured from the Herston Medical Research Centre (Brisbane, QLD, Australia) of The University of Queensland. On arrival at our facility, rats were approximately 3–4 weeks of age with a body weight in the range of ~50–70 g. Rats were caged in groups of two to three in a room with controlled temperature (23 ± 2°C) and a 12 h/12 h light–dark cycle. Standard rodent chow (Specialty Feeds, Glen Forrest, WA, Australia) and tap water were available to the rats *ad libitum*. Kimwipes (Kimberly-Clark Professional, Kirribilli, NSW, Australia) and Rat Chewsticks (Able Scientific, Canning Vale, WA, Australia) were provided as environmental enrichment. Rats were subject to acclimatization for at least 3 days prior to initiating experimentation. All the experiments herein were performed in the light phase. Approval of experimental procedures was obtained from the Animal Ethics Committee of The University of Queensland (Brisbane, QLD, Australia). The experiments were undertaken in accordance with the requirements of the Australian Code of Practice for the Care and Use of Animals for Scientific Purposes (8th edition, 2013).

### Surgical Procedure

Unilateral ITIs were performed in a manner similar to that described by others (Mao-Ying et al., 2006; Muralidharan et al., 2013) but with some modifications. Briefly, rats (~80–120 g) were anesthetized deeply with 3% isoflurane delivered in oxygen. Eye ointment was used to avoid drying of eyes during the surgical procedure. Benzylpenicillin injection was subcutaneously administered at a dose of 60 mg per rat. A unilateral rostro-caudal incision of approximately 1 cm in length was created on the upper medial half of the lower left hind limb. After the exposure of the tibia, using a 23-gauge needle, the bone was pierced medial to the tibial tuberosity below the knee joint. A 10 μL injection containing W256 cells in the required number, or HK cells (sham-rats) or DPBS (control rats) was administered into the bone cavity with a Hamilton^®^ syringe [80508:705SN 50 μL SYR SPECIAL (22/2″/4), Reno, NV, United States]. In all of the experiments (except experiment 4), at least one rat received an ITI of HK cells as a control for lack of pain hypersensitivity, including the experiments designed for pharmacological testing (Supplementary Table 1). The bone was immediately sealed using Ethicon^TM^ W810 bone wax (Johnson-Johnson International, Diegem, Belgium). The muscles and the skin were stitched in place with non-absorbable USP 5/0 sutures Dysilk^®^ suture (Dynek Pty Ltd, Hendon, SA, Australia). Topical antibiotic powder was then dusted on the wound. The hindpaw of the injected limb is termed as the ‘ipsilateral’ hindpaw, while that of the non-injected limb is termed as the ‘contralateral’ hindpaw. After the surgery, rats were closely monitored. The general animal health and their body weights were regularly assessed throughout the experimental period.

### Animal Groups and Experimental Timelines

**FIGURE 1:**
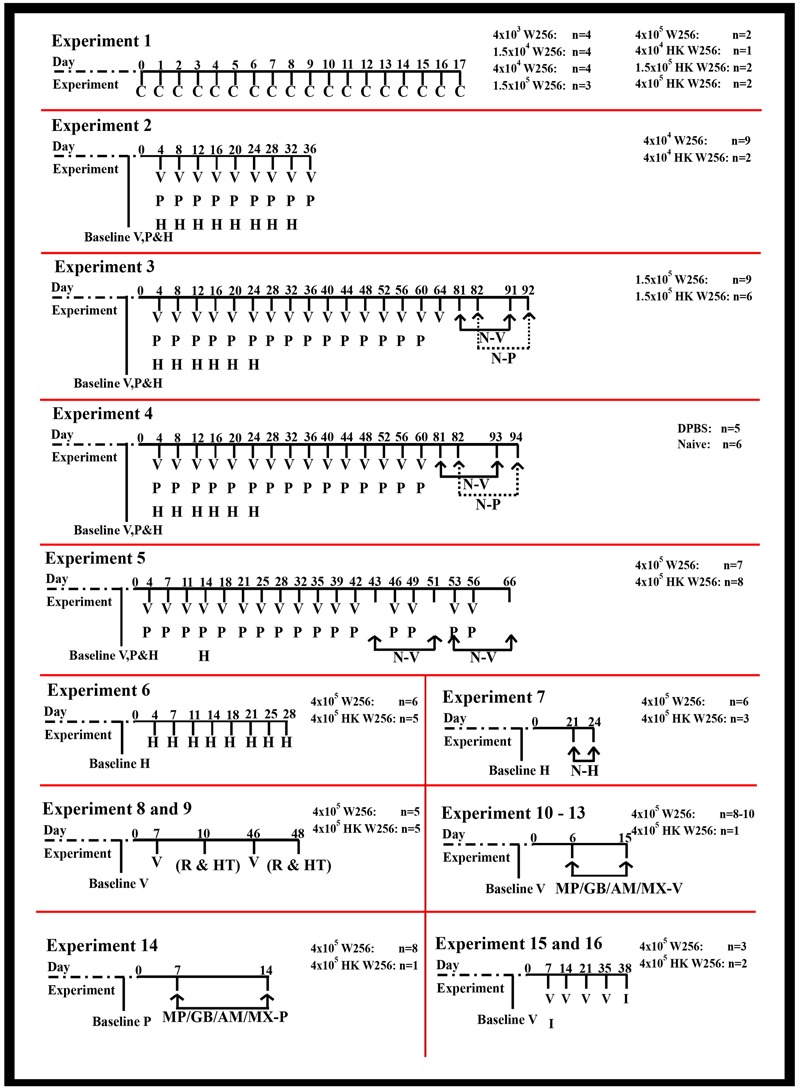
Timeline of assessments performed in individual experiments. AM, amitriptyline; C, clinical observations; GB, gabapentin; H, Hargreaves testing; HK, heat-killed; HT, histological assessment; I, immunohistochemical assessment; MP, morphine; MX, meloxicam; MP/GB/AM/MX-P, paw pressure testing after drug injection; MP/GB/AM/MX-V, von Frey testing after drug injection; N-H, Hargreaves testing after naloxone injection; N-P, paw pressure testing after naloxone injection; N-V, von Frey testing after naloxone injection; P, paw pressure testing; R, radiological assessment; V, von Frey testing; W256, Walker 256.

The number of animals involved in individual groups along with the details of each of the experiments performed together with their corresponding durations are shown in **Figure 1**. For the ease of editing and typesetting the figure, clinical observations have not been denoted for experiments 2–16 in **Figure 1**, although they were performed. Baseline pain behavioral tests prior to surgeries/ITI (referred as day 0) were performed on rats from all the experiments (Supplementary Table 2). To comprehensively characterize the model, one group of rats received an ITI of DPBS and another group (naïve) of animals did not undergo surgery or ITI (experiment 4).

### General Health Characteristics

The primary aim of Experiment 1 was to perform clinical observations and document temporal changes in general health along with body weights for the study duration. Animal welfare checks for mortality, morbidity and food and water levels were performed twice daily until the study completion. Body weights and clinical observations (general health parameters) were generally monitored at least once per week until the study completion in all the experiments. The observation method and the clinical parameters/symptoms were obtained and modified from the previously established standard methods (Moser and MacPhail, 1990; Haggerty, 1991) and Guidance Document on the Recognition, Assessment, and Use of Clinical Signs as Humane Endpoints for Experimental Animals Used in Safety Evaluation (OECD Environmental Health and Safety Publications Series on Testing and Assessment No. 19, 2000). Briefly, each rat in the study was carefully examined for their appearance, clonic movements, tonic movements, gait, stereotypy and bizarre behavior, if any. Assessment of appearance included parameters such as diarrhea, piloerection, salivation, palpebral closure, lacrimation, lack of grooming, bleeding from orifices and moribundity. Assessment of clonic movements included parameters such as wet dog shakes, clonic convulsions, myoclonic jerks, severe or whole body tremors, mild tremors, quivers of limbs, skin, ears or head, repetitive movements of jaws and mouth. Assessment of tonic movements included severe clonic and/or tonic convulsions causing dyspnoea, postictal depression or death, jumps with all feet leaving the surface, rigid forward extension of head and body, head and body rigidly arched backward and contraction of extensors. Assessment of gait included lameness, body drags, tiptoe walk, dragging or extension of forelimbs or inability to support weight, feet markedly point outward from the body, exaggerated or over-compensated movements of hind limbs, excessive sway, rocks or lurches and ataxia. Assessment of stereotypy involved repetitive sniffing, pacing, stereotypic grooming and circling. Assessment of bizarre behavior involved writhing or flopping, retropulsion, straub tail, self-mutilation and head weaving amongst others. The form used for recording clinical observations in experimental rats is shown in Supplementary Figure 1.

### Pain Behavioral Studies

#### Assessment of Mechanical Allodynia in the Hindpaws

Assessment of development of mechanical allodynia (hypersensitivity to applied non-noxious mechanical stimuli) in both hindpaws was performed using calibrated von Frey filaments (Stoelting Co., Wood Dale, IL, United States) by determining the lowest mechanical threshold that elicits a paw withdrawal response (Ren, 1999). Rats were individually positioned into wire mesh cages and they were acclimatized for around 15–30 min before von Frey testing. Until the filaments buckled slightly, they were physically applied on the plantar surface of the hindpaws. Absence of a response after 3 s suggested the use of a higher filament in the ascending order (2, 4, 6, 8, 10, 12, 14, 16, 18, and 20 g) until the response was observed. By contrast, a withdrawal response observed within 3 s suggested the use of a filament evoking a lower force than before. Testing was initiated with a 6 g filament, and the filaments were subsequently changed to obtain an increased or decreased force based upon the earlier response. The baseline PWTs for individual hindpaws were the mean of three observed readings with an interval of 5 min between two successive readings. For pharmacological testing of compounds at regular intervals over a 3-h post-dosing period, the starting filament at each time point was dependent upon the previous reading. Rats with PWTs ≤ 6 g in the ipsilateral hindpaw were defined as having fully developed mechanical allodynia. All of the von Frey assessments were performed in a blinded manner.

#### Assessment of Mechanical Hyperalgesia in the Hindpaws

Temporal development of mechanical hyperalgesia (hypersensitivity to applied noxious mechanical stimuli) in the bilateral hindpaws was determined using an Analgesy-meter (Ugo Basile, Italy). The mechanical force required to elicit withdrawal of each of the hindpaws (PPTs) of the animal was measured (Randall et al., 1957). Specifically, each hindpaw was positioned on a small plinth under a rounded cone-shaped pusher which avoids tissue damage. Depressing the pedal-switch started the mechanism of exertion of force. As soon as the rat struggled, the pedal was immediately released and the applied force was recorded. A cut-off of 200 g maximum was used to avoid hindpaw injury. Baseline PPTs were the mean of three observations for a hindpaw with an interval of at least 5-min between consecutive assessments. Rats with ipsilateral PPTs ≤ 80 g were considered to have fully developed mechanical hyperalgesia. All of the PPT assessments were performed in a blinded manner.

#### Assessment of Thermal Hyperalgesia in the Hindpaws

Development of thermal hyperalgesia (hypersensitivity to applied noxious heat stimuli) in the hindpaws was studied using the plantar test/Hargreaves apparatus (Ugo Basile, Italy) to assess the time required to elicit a withdrawal response (PTTs) to an applied noxious heat stimulus (Hargreaves et al., 1988). Rats were individually positioned in Perspex chambers having a glass floor and acclimatized for at least 30 min before performing the test. A noxious heat stimulus [infrared (IR) intensity value-30; 135 mW/cm^2^] was radiated to the plantar surface of the hindpaws and the PTT values were noted. A cut-off time of a maximum of 30 s was used to avoid damage to the tissue. Baseline PTTs were the mean of three observations for a hindpaw with an interval of 5-min between consecutive tests. All of the PTT assessments were performed in a blinded manner.

#### Test Compound Administration

Animals were dosed by the first person and assessments were performed by the second person to ensure that study blinding was not compromised. Each rat received a maximum of five intraperitoneal (i.p.) or subcutaneous (s.c.) doses of test compounds or vehicle with at least 2 days of ‘washout’ between consecutive doses. Test compound dosing solutions in the present work were prepared using Water for injection BP as the vehicle. Doses of all test compounds are expressed in terms of their water-soluble salts similar to previous studies from our laboratory (South et al., 2009; Varamini et al., 2012; Smith et al., 2013; Han et al., 2014; Kuo et al., 2015) and by others (Negus et al., 2012; Maguire et al., 2013; Samal et al., 2015).

##### Effect of naloxone on pain phenotypes

Rats from experiments 3, 4, 5, and 7 were given single bolus doses of naloxone (15 mg/kg s.c.) or vehicle after the apparent hypersensitivity of the hindpaws to applied mechanical stimuli had resolved. After injecting naloxone or vehicle, PWTs, PPTs or PTTs were assessed in the hindpaws at pre-defined intervals during a 3 h period post-dosing.

##### Anti-allodynic effect of morphine, gabapentin, amitriptyline and meloxicam

Rats having fully developed mechanical allodynia in their ipsilateral hindpaws were administered single bolus doses of morphine (0.3, 1, and 3 mg/kg s.c.), gabapentin (30, 70, and 100 mg/kg i.p.), amitriptyline (3, 10, and 30 mg/kg i.p.) and meloxicam (2.5, 5.0, and 7.5 mg/kg i.p.) or vehicle. PWTs were measured in both hindpaws immediately pre-dose and at 0.25, 0.5, 0.75, 1.0, 1.25, 1.5, 2, and 3 h post-dosing.

##### Anti-hyperalgesic effect of morphine, gabapentin, amitriptyline and meloxicam

Paw pressure thresholds were measured in both hindpaws of rats with fully developed mechanical hyperalgesia following administration of single bolus doses of morphine (3 mg/kg s.c.), gabapentin (100 mg/kg i.p.), amitriptyline (30 mg/kg i.p.), meloxicam (7.5 mg/kg i.p.) or vehicle. PPTs were assessed immediately before dosing and at 0.25, 0.5, 0.75, 1.0, 1.25, 1.5, 2, and 3 h post-dosing.

### Tibial Bone μCT Scan

Rats (ITI of 4 × 10^5^ W256 cells, *n* = 3; ITI of 4 × 10^5^ HK W256 cells, *n* = 3) from Experiments 8 and 9 were euthanized on day 10 and day 48 with an overdose of pentobarbitone sodium (1 mL/kg of 325.73 g/L; Lethabarb^®^). The tibiae were collected and fixed in 10% NBF for at least 2 days. Micro-CT scanning was conducted using a preclinical Inveon Multimodality PET/CT Scanner (Siemens Medical Soln., Knoxville, TN, United States) at the Centre for Advanced Imaging (CAI) at The University of Queensland. The μ-CT images were obtained using Inveon Acquisition Workstation software (IAW version 2.0, Siemens). The X-ray source voltage was set to 80 kV and the current to 250 μA. The scans were conducted using 360° rotation with 360 rotation steps using a medium–high magnification and with a binning factor of 2. The exposure time was 2300 ms and the CT scanning process totally took approximately 60 min. The μ-CT images were reconstructed using a Feldkamp reconstruction software (Siemens) resulting in an isotropic voxel dimension of 27.9 μm. The CT data were calibrated in Hounsfield units (HU) defined such that the water and air have 0 and 1000 HU values, respectively. The images were analyzed using Inveon Research Workstation software (IRW version 4.1, Siemens) to measure the bone volume/total volume (BV/TV ratio), trabecular thickness (T_b_.T_h_), trabecular spacing (T_b_.S_p_) and trabecular number (T_b_.N) in the proximal diaphyseal regions of the ipsilateral tibial bones, as described previously (Muralidharan et al., 2013).

### Tibial Bone Histology

Rats (ITI of 4 × 10^5^ W256 cells, *n* = 3; ITI of 4 × 10^5^ HK W256 cells, *n* = 3) from Experiments 8 and 9 were euthanized on day 10 and day 48 with pentobarbitone sodium (1 mL/kg of 325.73 g/L; Lethabarb^®^). Tibiae were harvested and fixed by immersing in 10% NBF (Jin et al., 2016; Liu et al., 2016) for at least 2 days. These tibiae were then immersed in 15% w/v solution of UltraPure^TM^ EDTA in phosphate buffer for at least 4 weeks, with the EDTA solution being changed twice per week (Hald et al., 2009). The soft decalcified bones were then rinsed, and after dehydration they were embedded in paraffin and cut into 4 μm cross-sections with a RM2235 rotary microtome (Leica Microsystems, Wetzlar, Germany) at the QIMR Berghofer Medical Research Institute, Brisbane, QLD, Australia. Sections of proximal diaphyseal regions of ipsilateral tibiae were mounted on Uber slides (InstrumeC Pty Ltd., Beaumaris, VIC, Australia) and stained using hematoxylin and eosin (H&E) (Mao-Ying et al., 2006) to assess histological changes in the tibial structure.

### Immunocytochemistry of W256 Cells: Cytokeratin 18

W256 cells were seeded onto sterile coverslips in 24 well-plates. Once the cells were 80–90% confluent, the culture medium was aspirated and the cells were briefly rinsed using PBS. The cells were fixed with ice-cold methanol (4 min at -20°C). The fixing agent was aspirated and the cells were washed with 0.2% Tween 20 and 0.1% Triton^TM^ X-100 in PBS for 10 min. The fixed cells were subsequently blocked for 30 min with 1% BSA in PBS at 23 ± 2°C. Anti-Cytokeratin 18 antibody [C04] (Alexa Fluor^®^ 488) ab187573 (1:20 dilution, Abcam, Melbourne, VIC, Australia), prepared in 1% BSA in PBS, was added to the wells containing the coverslips and the plate was incubated at 37°C for 3 h in the dark (~0.002 lux). The antibody solution in the wells was then aspirated and the cells were rinsed with PBS thrice for 5 min each. A 0.5 μg/mL solution of DAPI was added and the cells were incubated for 5–10 min. The DAPI solution was then aspirated and the cells were washed with PBS. Next, the processed cells on coverslips were mounted on Superfrost^®^ Plus slides (Lomb Scientific Pty Ltd., Taren Point, NSW, Australia) using Prolong^®^ Gold antifade reagent. The mounted slides were allowed to dry and stabilize in the dark at 4–8°C overnight, prior to image capture using a fluorescence microscope as mentioned in Section Image Acquisition.

To validate the above results of immunocytochemical staining, anti-Cytokeratin 18 antibody [C-04] ab668 (1:100 dilution, Abcam, Melbourne, VIC, Australia) was used as a second confirmatory antibody, because previous works published in *Nature* (Leong et al., 2008; Wang et al., 2009) have validated the suitability of this antibody in both immunocytochemistry and immunohistochemistry applications. The procedure for staining of cells with the second antibody was similar to that of the previous antibody as described above. However, after the primary antibody treatment, the cells were incubated with Goat anti-Mouse IgG (H+L) Secondary Antibody, Alexa Fluor 546 A-11030 (1:500 dilution, Thermo Fisher Scientific, Rockford, IL, United States) in PBS with 0.1% Tween 20 (PBST) for around 2 h in the dark (~0.002 lux) at 23 ± 2°C, followed by 2 × 5 min washes with PBST and 1 × 5 min wash with PBS before treatment with DAPI.

### Tibial Bone Immunohistochemistry

Rats (ITI of 4 × 10^5^ W256 cells, *n* = 3; ITI of 4 × 10^5^ HK W256 cells, *n* = 2) from experiment 15 and 16 were euthanized on days 7 and 38 with an overdose of pentobarbitone and then perfusion-fixed using 4% PFA. The tibiae were collected and further post-fixed with 4% PFA. The tibiae were stored in 10% NBF for 2 days at 4–8°C. These tibiae were then decalcified in a manner similar to that described in Section Tibial Bone Histology. The decalcified bones were allowed to post-fix for 2 h in 4% PFA solution (4–8°C), cryoprotected successively in 15% sucrose/PBS and 30% sucrose/PBS at 4–8°C and subsequently placed in a 1:1 mixture of OCT:30% sucrose/PBS at 4–8°C, after which they were freeze-mounted in Tissue-Tek^®^ O.C.T. Compound. Frozen tibial longitudinal sections (7 μm thick) were cut using a Cryostar NX70, (Thermo Fisher Scientific, Waltham, MA, United States) and mounted on Uber Plus charged slides (InstrumeC, Beaumaris, VIC, Australia). In order to identify the tumor infiltration by using immunohistochemistry, tibial sections were washed with a 1× PBS (pH 7.4) solution (3 × 5 min), followed by blocking with 10% NGS containing 0.3% Triton^TM^ X-100 in PBS for 1–2 h at room temperature. The sections were allowed to incubate with anti-Cytokeratin 18 antibody [C04] (Alexa Fluor^®^ 488) ab187573 (1:20 dilution) diluted in 2% NGS in PBST overnight at 4–8°C. This was followed by 2 × 5 min washes using PBST and 1 × 5 min wash with PBS. The sections were allowed to incubate with DAPI for 5–10 min and subsequently washed with PBS (2 × 5 min) and the cover-slips were placed along with Prolong^®^ Gold antifade reagent.

Similar to the procedure outlined in Section Immunocytochemistry of W256 Cells: Cytokeratin 18, anti-Cytokeratin 18 antibody [C-04] ab668 (1:100 dilution) was also used to validate the results of immunohistochemical staining. The procedure for staining of tibial sections with the second antibody was similar to that of the previous antibody described above. However, the sections were allowed to incubate with primary antibody overnight at 23 ± 2°C, and then incubated with Goat anti-Mouse IgG (H+L) Secondary Antibody, Alexa Fluor 546 A-11030 (1:600 dilution) in PBST for 2 h in the dark (~0.002 lux) at 23 ± 2°C. This was followed by 2 × 5 min washes using PBST and 1 × 5 min wash with PBS and the sections were subsequently treated with DAPI and mounted as described above.

### Image Acquisition

For the histology experiments, images were captured with an Aperio ScanScope XT system (Leica Biosystems, Nussloch, Germany) located at the School of Biomedical Sciences, The University of Queensland and processed using Aperio ImageScope v12.3.0.5056 software (Leica Biosystems). Images from immunocytochemistry and immunohistochemistry experiments were captured with an Axioskop 40 microscope (Carl Zeiss, Göttingen, Germany) attached to an Axiocam MRm camera (Carl Zeiss) and processed using AxioVision Rel. v4.8 software (Carl Zeiss). For the immunocytochemistry and immunohistochemistry experiments, images were acquired at a fixed exposure time, optimized using auto-exposure settings of AxioVision Rel. v4.8 software and by using filters suitable for the fluorophore of the secondary antibody.

### Data Analysis

All the values have been expressed as mean ± standard error of the mean (SEM). For treatments having *n* ≤ 2, the values are expressed as mean or absolute value, without calculating the error. Generation of graphs and data processing were done using the GraphPad Prism^TM^ (v7.00) software package. The PWT values of rats administered single bolus doses of test compound or vehicle were normalized by subtracting the pre-dosing baseline values so as to obtain ΔPWT values as follows:

ΔPWT value=(postdosing PWT)−(Average predosing baseline PWT)

Area under the curve (AUC) for ΔPWT versus time curves (ΔPWT AUC values) was calculated using trapezoidal integration to determine the extent and duration of action of test compounds for relief of allodynia. ΔPWT AUC values were then converted into a percentage of the maximum possible ΔPWT AUC (% MAX ΔPWT AUC) with the following formula:

$$\%\text{MAX }\Delta \text{PWT AUC}=\frac{\Delta \text{PWT AUC}}{\text{Maximum }\Delta \text{PWT AUC}}\times 100$$

Dose–response curves were generated by plotting mean (±SEM) % MAX ΔPWT AUC values versus log dose of each of the test compounds. The PPT data and the naloxone data were processed in a similar way as described above. Non-linear regression (GraphPad Prism^TM^ v7.00) was used to determine the ED_50_ values for each drug treatment against mechanical allodynia in the ipsilateral as well as the contralateral hindpaws.

### Statistical Analysis

Statistical analyses were performed using the GraphPad Prism^TM^ v7.00 software package. The criterion of statistical significance was *p* ≤ 0.05. Two-way analysis of variance (ANOVA) followed by the Bonferroni test was used to analyze between-group differences in body weights, behavioral data, pharmacological data and tibial bone morphometric changes. The Mann–Whitney test was used to assess between group body weight differences with missing intermediate values and to compare differences in ΔPPT AUC values for each test compound- and vehicle-treated group. One-way ANOVA followed by the Dunnett’s test was used in order to compare differences in ΔPWT AUC values for each test compound- and vehicle-treated groups. The unpaired *t*-test was used in order to compare the extent and duration of naloxone induced rescue of the pain phenotype. For statistical comparisons using ANOVA, *F*-values were reported along with their associated degrees of freedom (treatment, time, interaction and residual). For two-way ANOVA, *F*-values were expressed as F(df of treatment, time, interaction/residual). Whereas, for one-way ANOVA, *F*-values were expressed as F(df of treatment, residual).

## Results

### General Health Characteristics

**FIGURE 2:**
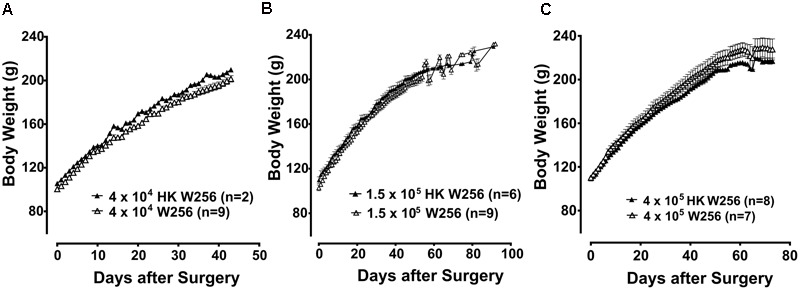
Body weight of rats from individual experiments. Panels in the figure show mean (±SEM) body weight of rats from **(A)** experiment 2, **(B)** experiment 3 and **(C)** experiment 5. HK, heat-killed; W256, Walker 256. There were no statistically significant differences in body weight between any treatment groups (*p* > 0.05; experiment 2, Two-way ANOVA, *post hoc* Bonferroni test; experiment 3 and 5, Mann–Whitney test).

In terms of body weight, there were no significant differences between animals given an ITI of W256 and HK W256 cells in experiment 1 [*F*_(7,17,119/238)_ = 1.2, 1125, 1.5; *p* > 0.05], experiment 2 [*F*_(1,43,43/387)_ = 2.2, 619.7, 0.9; *p* > 0.05] (**Figure 2A**), experiment 3 (*p* > 0.05) (**Figure 2B**), experiment 5 (*p* > 0.05) (**Figure 2C**), experiment 7 [*F*_(1,14,14/98)_ = 0.7, 766.6, 1.3; *p* > 0.05], experiment 8 [*F*_(1,8,18/64)_ = 0.4, 400.8, 2.7; *p* > 0.05], experiment 9 [*F*_(1,11,11/88)_ = 1.1, 364, 0.05; *p* > 0.05], experiment 10 [*F*_(1,8,8/64)_ = 0.3, 180.6, 2.8; *p* > 0.05], experiment 11 [*F*_(1,8,8/64)_ = 1.0, 314.8, 1.6; *p* > 0.05], experiment 12 [*F*_(1,7,7/63)_ = 0.5, 66.0, 0.2; *p* > 0.05], experiment 13 [*F*_(1,7,7/49)_ = 1.1, 195.4, 0.8; *p* > 0.05] and experiment 14 [*F*_(1,9,9/63)_ = 0.7, 141.5, 1.6; *p* > 0.05]. Additionally, there were no significant differences in between body weights of animals given an ITI of DPBS and age-matched control female Wistar Han rats in experiment 4 (*p* > 0.05). Although there were slight differences between body weights of rats administered W256 and HK W256 cells in experiment 6 [*F*_(1,12,12/108)_ = 8.9, 508.1, 3.1; *p* ≤ 0.05], experiment 15 [*F*_(1,6,6/18)_= 11.9, 84.1, 1.3; *p* ≤ 0.05] and experiment 16 [*F*_(1,11,11/33)_= 62.6, 314.2, 0.6; *p* ≤ 0.05] at some time points, these were either due to differences in initial body weights or due to non-cancer factors. Importantly, there were consistent gains in the body weights of all animals throughout the experiments (Supplementary Figure 2).

A total of 173 rats were used in this study, out of which 9 rats (5%) were found to have health-related issues and of these 3 (1.7%) were euthanized for ethical reasons as outlined below. One rat from experiment 1 given an ITI of 4 × 10^3^ W256 cells had a slight tumor like appearance in the bone near the injection site. Nevertheless, the overall health of the animal was satisfactory and no other complications were observed. Another rat from experiment 1 given an ITI of 1.5 × 10^5^ W256 cells had a mild infection at the sight of surgery with visible pus exudate. Under anesthesia, the infected site was cleaned and topical antibiotic powder was applied, followed by a s.c. injection of benzylpenicillin at a dose of 60 mg. Subsequently, the infection resolved. One rat given an ITI of 4 × 10^3^ W256 cells, two rats given an ITI of 1.5 × 10^5^ W256 cells and one rat given an ITI of 4 × 10^5^ HK W256 cells from experiment 1 had mild swelling at the injection site, which resolved spontaneously. One rat from experiment 4 given an ITI of DPBS was excluded from the study and euthanized due to lack of normal body weight gain due to a crooked incisor. One rat given an ITI of 4 × 10^5^ W256 cells from experiment 5 was excluded and euthanized due to the presence of a large external tumor growth on the injected tibia. One rat from experiment 6 given an ITI of 4 × 10^5^ W256 cells was excluded and euthanized due to lethargic behavior and low body weight; upon post-mortem investigation, a large metastatic lesion was observed on the intestine.

### Assessment of Mechanical Allodynia in the Hindpaws

#### Experiment 2

**FIGURE 3:**
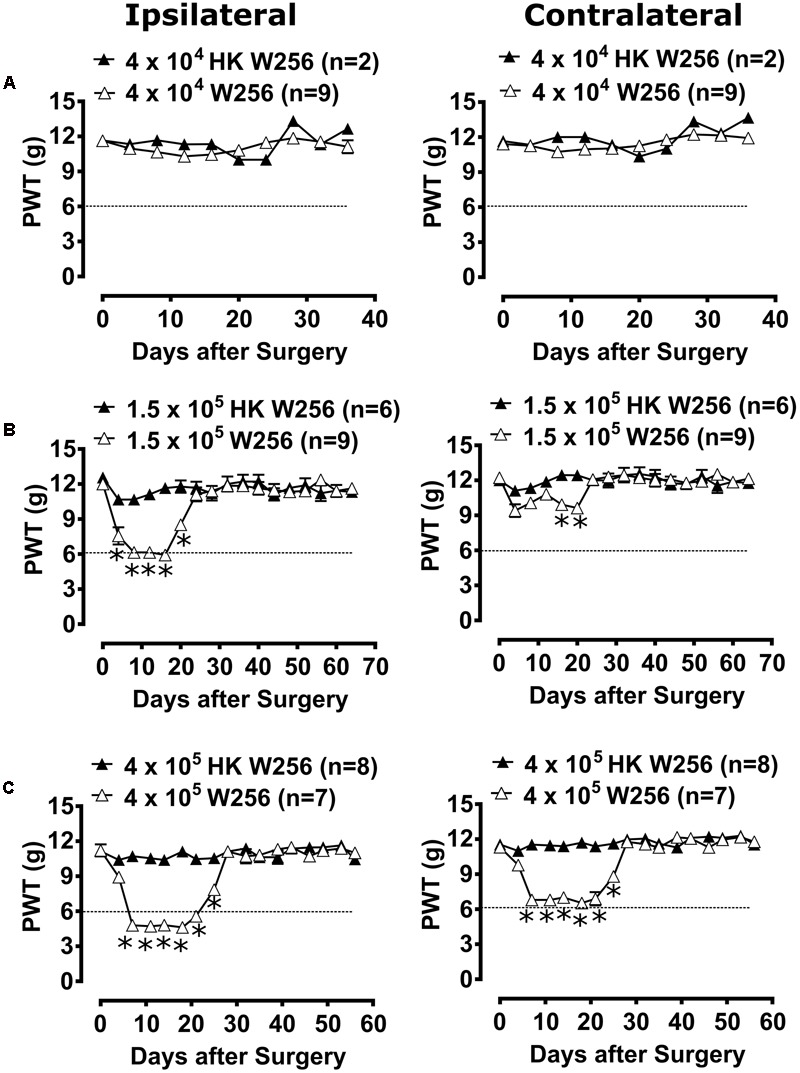
Paw withdrawal thresholds (PWTs) of ipsilateral and contralateral hindpaws of rats. Panels in the figure show mean (±SEM) PWTs of rats from **(A)** experiment 2, **(B)** experiment 3 and **(C)** experiment 5. Rats with PWTs ≤6 g in the ipsilateral hindpaw were considered to have fully developed mechanical allodynia as indicated by the dotted line. HK, heat-killed; W256, Walker 256. ^∗^*p* ≤ 0.05 (Two-way ANOVA, *post hoc* Bonferroni test) *c.f.* rats given an ITI of HK W256 cells.

Unilateral ITI of 4 × 10^4^ W256 cells in rats did not significantly reduce PWTs in either the ipsilateral [*F*_(1,9,9/81)_= 0.8, 2.1, 1.3; *p* > 0.05] or the contralateral [*F*_(1,9,9/81)_ = 4.2, 2.9, 1.2; *p* > 0.05] hindpaws throughout the experiment *c.f.* rats administered a unilateral ITI of 4 × 10^4^ HK W256 cells (**Figure 3A**).

#### Experiment 3

Unilateral ITI of 1.5 × 10^5^ W256 cells in rats significantly reduced the PWTs in the ipsilateral [*F*_(1,16,16/208)_ = 66.9, 13.4, 8.6; *p* ≤ 0.05] hindpaw between days 4 and 20 after surgery and in the contralateral [*F*_(1,16,16/208)_= 14.7, 3.3, 2.4; *p* ≤ 0.05] hindpaws between days 16 and 20 after surgery *c.f.* rats given an ITI of 1.5 × 10^5^ HK W256 cells, with a maximum reduction of 50.6 and 23.0% in the ipsilateral and contralateral PWTs relative to the corresponding baseline PWTs, respectively (**Figure 3B**).

#### Experiment 4

Unilateral ITI of DPBS in rats did not significantly reduce PWTs in either the ipsilateral [*F*_(1,15,15/135)_= 0.4, 1.9, 1.2; *p* > 0.05] or the contralateral [*F*_(1,15,15/135)_ = 0.4, 1.6, 1.1; *p* > 0.05] hindpaws throughout the experiment *c.f.* age-matched control (naïve) rats (Supplementary Figure 3A).

#### Experiment 5

Unilateral ITI of 4 × 10^5^ W256 cells in rats significantly reduced the PWTs in both the ipsilateral [*F*_(1,16,16/208)_ = 228.0, 35.5, 25.8; *p* ≤ 0.05] and contralateral [*F*_(1,16,16/208)_= 154.4, 28.0, 20.5; *p* ≤ 0.05] hindpaws between days 7 and 25 after surgery *c.f.* rats given an ITI of 4 × 10^5^ HK W256 cells, with a maximum reduction of 58.9 and 42.3% in the ipsilateral and contralateral PWTs relative to the corresponding baseline PWTs, respectively (**Figure 3C**).

#### Experiment 8

Unilateral ITI of 4 × 10^5^ W256 cells in rats significantly reduced the PWTs in both the ipsilateral [*F*_(1,1,1/8)_= 81.6, 140.5, 45.5; *p* ≤ 0.05] and contralateral [*F*_(1,1,1/8)_= 39.6, 103.7, 55.8; *p* ≤ 0.05] hindpaws at day 7 after surgery *c.f.* rats given a unilateral ITI of 4 × 10^5^ HK W256 cells, with an observed decrease of 58.8 and 51.3% in the ipsilateral and contralateral PWTs relative to the corresponding baseline PWTs, respectively (Supplementary Figure 3B).

#### Experiment 9

Unilateral ITI of 4 × 10^5^ W256 cells in rats significantly reduced the PWTs in both the ipsilateral [*F*_(1,2,2/16)_= 26.1, 79.3, 41.9; *p* ≤ 0.05] and contralateral [*F*_(1,2,2/16)_= 25.2, 47.9, 26.6; *p* ≤ 0.05] hindpaws at day 7 after surgery *c.f.* rats administered a unilateral ITI of 4 × 10^5^ HK W256 cells, with a reduction of 58.6 and 52% in the ipsilateral and contralateral PWTs, respectively. At day 46 after surgery there was no significant difference between PWTs of both ipsilateral [*F*_(1,2,2/16)_= 26.1, 79.3, 41.9; *p* > 0.05] and contralateral [*F*_(1,2,2/16)_ = 25.2, 47.9, 26.6; *p* > 0.05] hindpaws *c.f.* rats administered a unilateral ITI of 4 × 10^5^ HK W256 cells (Supplementary Figure 3C).

#### Experiment 15

Unilateral ITI of 4 × 10^5^ W256 cells in rats significantly reduced the PWTs in both the ipsilateral [*F*_(1,1,1/3)_ = 171.6, 25.4, 15.0; *p* ≤ 0.05] and contralateral [*F*_(1,1,1/3)_= 95.3, 28.9, 28.9; *p* ≤ 0.05] hindpaws at day 7 after surgery *c.f.* rats administered a unilateral ITI of 4 × 10^5^ HK W256 cells, with a reduction of 52.2 and 27.9% in the ipsilateral and contralateral PWTs, respectively (Supplementary Figure 3D).

#### Experiment 16

Unilateral ITI of 4 × 10^5^ W256 cells in rats significantly reduced the PWTs in both the ipsilateral [*F*_(1,4,4/12)_= 98.7, 13.2, 12.7; *p* ≤ 0.05] and contralateral [*F*_(1,4,4/12)_= 67.6, 6.0, 5.1; *p* ≤ 0.05] hindpaws between days 7 and 21 after surgery *c.f.* rats administered a unilateral ITI of 4 × 10^5^ HK W256 cells, with a maximum reduction of 55.6 and 33.3% in the ipsilateral and contralateral PWTs relative to the corresponding baseline PWTs, respectively (Supplementary Figure 3E).

### Assessment of Mechanical Hyperalgesia in the Hindpaws

#### Experiment 2

**FIGURE 4:**
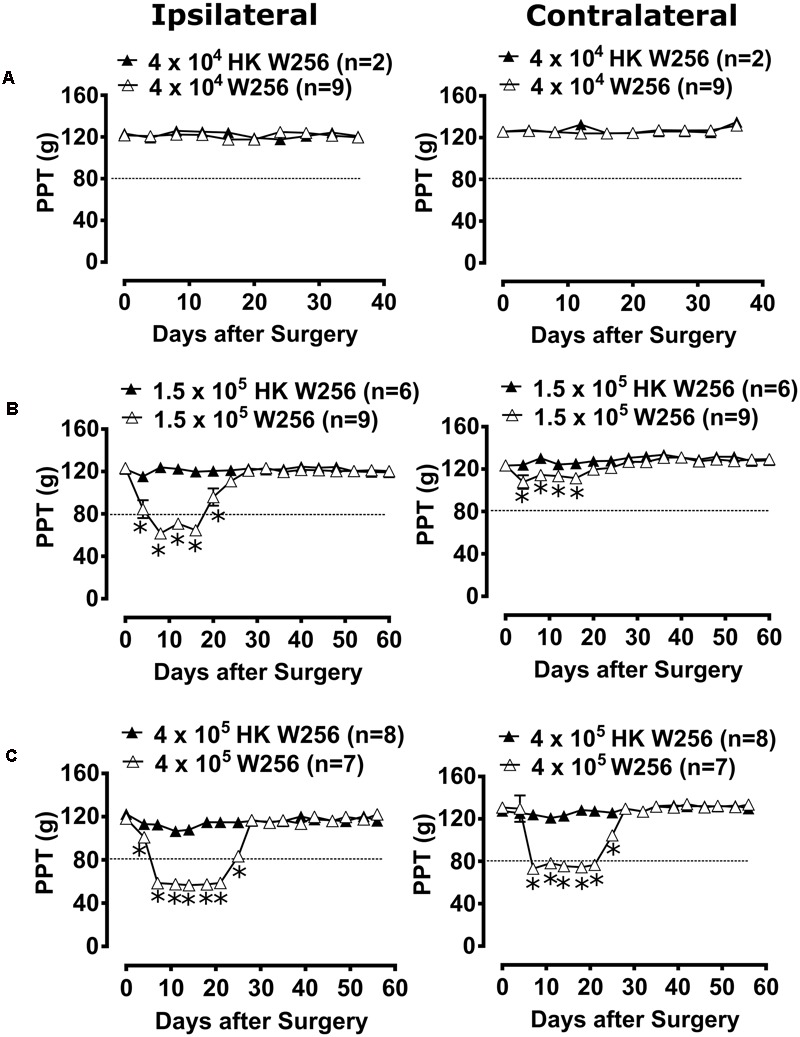
Paw pressure thresholds (PPTs) of ipsilateral and contralateral hindpaws of rats. Panels in the figure show mean (±SEM) PPTs of rats from **(A)** experiment 2, **(B)** experiment 3 and **(C)** experiment 5. Rats with PPTs ≤80 g in the ipsilateral hindpaw were considered to have fully developed mechanical hyperalgesia as indicated by the dotted line. HK, heat-killed; W256, Walker 256. ^∗^*p* ≤ 0.05 (Two-way ANOVA, *post hoc* Bonferroni test) *c.f.* rats given an ITI of HK W256 cells.

Unilateral ITI of 4 × 10^4^ W256 cells in rats did not significantly reduce PPTs in either the ipsilateral [*F*_(1,9,9/81)_= 0.1, 0.5, 0.7; *p* > 0.05] or the contralateral [*F*_(1,9,9/81)_ = 0.1, 1.5, 0.5; *p* > 0.05] hindpaws throughout the experiment *c.f.* rats administered a unilateral ITI of 4 × 10^4^ HK W256 cells (**Figure 4A**).

#### Experiment 3

Unilateral ITI of 1.5 × 10^5^ W256 cells in rats significantly reduced the PPTs in the ipsilateral [*F*_(1,15,15/195)_ = 185.4, 22.0, 20.4; *p* ≤ 0.05] hindpaws between days 4 and 20 after surgery and in the contralateral [*F*_(1,15,15/195)_= 68.0, 7.5, 2.6; *p* ≤ 0.05] hindpaws between days 4 and 16 after surgery *c.f.* rats administered a unilateral ITI of 1.5 × 10^5^ HK W256 cells, with a maximum reduction of 50 and 12.7%, respectively, in the ipsilateral and the contralateral PPTs relative to corresponding baseline PPTs (**Figure 4B**).

#### Experiment 4

Unilateral ITI of DPBS in rats did not significantly reduce PPTs in either the ipsilateral [*F*_(1,15,15/135)_= 0.001, 2.3, 1.6; *p* > 0.05] or the contralateral [*F*_(1,15,15/135)_ = 1.3, 3.1, 1.3; *p* > 0.05] hindpaws throughout the experiment *c.f.* naïve non-injected rats (Supplementary Figure 4).

#### Experiment 5

Unilateral ITI of 4 × 10^5^ W256 cells in rats significantly reduced the PPTs in the ipsilateral [*F*_(1,16,16/208)_ = 1262, 137.6, 91.8; *p* ≤ 0.05] hindpaws between days 4 and 25 after surgery and in the contralateral [*F*_(1,16,16/208)_= 203.7, 55.4, 37.1; *p* ≤ 0.05] hindpaws between days 7 and 25 *c.f.* rats administered a unilateral ITI of 4 × 10^5^ HK W256 cells, with a maximum reduction of 52.1 and 44.1% in the ipsilateral and the contralateral PPTs relative to the corresponding baseline PPTs, respectively (**Figure 4C**).

### Assessment of Thermal Hyperalgesia in the Hindpaws

#### Experiment 2

**FIGURE 5:**
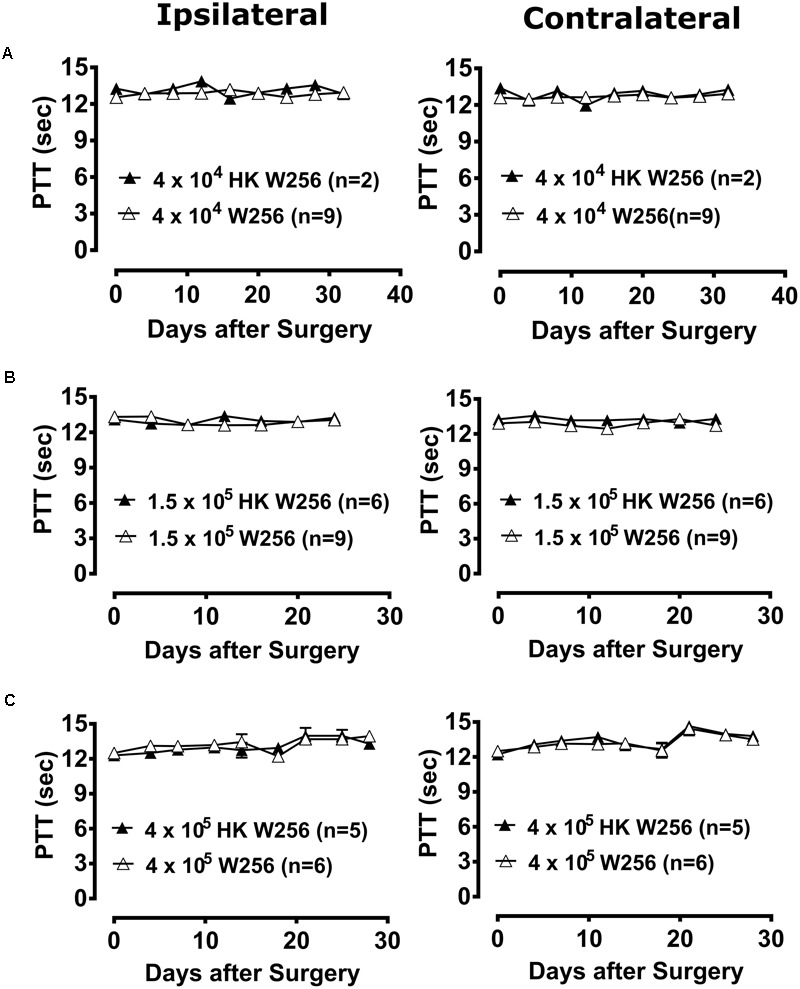
Paw thermal thresholds (PTTs) of ipsilateral and contralateral hindpaws of rats. Panels in the figure show mean (±SEM) PTTs of rats from **(A)** experiment 2, **(B)** experiment 3 and **(C)** experiment 6. HK, heat-killed; W256, Walker 256. There were no statistically significant differences in PTTs between the treatment groups in any of these experiments (*p* > 0.05; Two-way ANOVA, *post hoc* Bonferroni test).

Unilateral ITI of 4 × 10^4^ W256 cells in rats did not significantly reduce PTTs in either the ipsilateral [*F*_(1,8,8/72)_= 2.7, 0.5, 1.1; *p* > 0.05] or the contralateral [*F*_(1,8,8/72)_ = 0.3, 1.1, 0.6; *p* > 0.05] hindpaws throughout the experiment *c.f.* rats administered a unilateral ITI of 4 × 10^4^ HK W256 cells (**Figure 5A**).

#### Experiment 3

Unilateral ITI of 1.5 × 10^5^ W256 cells in rats did not significantly reduce PTTs in either the ipsilateral [*F*_(1,6,6/78)_ = 0.4, 1.5, 1.8; *p* > 0.05] or the contralateral [*F*_(1,6,6/78)_ = 6.1, 0.9, 1.1; *p* > 0.05] hindpaws throughout the experiment *c.f.* rats administered a unilateral ITI of 1.5 × 10^5^ HK W256 cells (**Figure 5B**).

#### Experiment 4

Unilateral ITI of DPBS in rats did not significantly reduce PTTs in either the ipsilateral [*F*_(1,6,6/54)_ = 0.1, 1.3, 2.5; *p* > 0.05] or the contralateral [*F*_(1,6,6/54)_ = 1.9, 0.6, 1.0; *p* > 0.05] hindpaws throughout the experiment *c.f.* naïve non-injected rats (Supplementary Figure 5A).

#### Experiment 5

Unilateral ITI of 4 × 10^5^ W256 cells in rats did not significantly reduce PTTs in either the ipsilateral [*F*_(1,1,1/13)_ = 0.0002, 0.1, 0.2; *p* > 0.05] or the contralateral [*F*_(1,1,1/13)_ = 1.1, 0.7, 0.7; *p* > 0.05] hindpaws at day 14 after surgery *c.f.* rats administered a unilateral ITI of 4 × 10^5^ HK W256 cells (Supplementary Figure 5B).

#### Experiment 6

Unilateral ITI of 4 × 10^5^ W256 cells in rats did not significantly reduce PTTs in either the ipsilateral [*F*_(1,8,8/72)_ = 1.0, 3.1, 0.7; *p* > 0.05] or the contralateral [*F*_(1,8,8/72)_ = 0.8, 6.7, 0.3; *p* > 0.05] hindpaws throughout the experiment *c.f.* rats administered a unilateral ITI of 4 × 10^5^ HK W256 cells (**Figure 5C**).

Based upon the cell number-dependent experimental results in female Wistar Han rats administered a unilateral ITI of W256 cells, 400,000 cells/10 μL DPBS was found to be the optimum cell number as it produced distinct bilateral hindpaw hypersensitivity whilst maintaining satisfactory animal health throughout the study (**Table 1**).

**Table 1:** Summary of W256 cell number-dependent variations in the experimental outcomes in female Wistar Han rats administered a unilateral ITI of these cells.

			Mechanical allodynia						Mechanical hyperalgesia						Thermal hyperalgesia	
			Ipsi			Contra			Ipsi			Contra			Ipsi	Contra
Number of cells injected	Gain in body weight	Animal health	Obs.	Dur. (days)	Ext. (%)	Obs.	Dur. (days)	Ext. (%)	Obs.	Dur. (days)	Ext. (%)	Obs.	Dur. (days)	Ext. (%)		
40,000	✓	✓	-	N/A	N/A	-	N/A	N/A	-	N/A	N/A	-	N/A	N/A	-	-
150,000	✓	✓	+	4–20	50.6	+	16–20	23.0	+	4–20	49.9	+	4–16	12.7	-	-
400,000	✓	✓	+	7–25	58.8	+	7–25	42.2	+	4–25	52.1	+	7–25	44.0	-	-

*✓, satisfactory; +, observed; -, not observed; Contra., contralateral hindpaws; Dur., duration in days post-ITI; Ext., extent of percentage reduction in PWTs/PPTs; Ipsi., ipsilateral hindpaws; N/A, not applicable; Obs., observation.*

### Test Compound Administration

#### Effect of Naloxone on Pain Behavioral Phenotypes

##### Experiment 3

Administration of naloxone in the time interval, day 81–91, to a group of rats administered a unilateral ITI of 1.5 × 10^5^ W256 cells significantly reduced the PWTs from 0.25 to 0.75 h after injection in both the ipsilateral [*F*_(1,8,8/104)_ = 12.8, 5.3, 1.1; *p* ≤ 0.05] and contralateral [*F*_(1,8,8/104)_= 9.6, 5.2, 2.8; *p* ≤ 0.05] hindpaws *c.f.* rats administered a unilateral ITI of 1.5 × 10^5^ HK W256 cells, with a maximum reduction of 24 and 26.4% in the ipsilateral and contralateral PWTs relative to the corresponding baseline PWTs, respectively (Supplementary Figure 6A). Administration of naloxone to these rats in the interval, day 82–92, significantly [*F*_(1,8,8/104)_= 3.1, 1.4, 4.3; *p* ≤ 0.05] reduced the PPTs from 0.75 to 1 h after injection in the ipsilateral hindpaw and significantly [*F*_(1,8,8/104)_= 3.7, 1.9, 2.3; *p* ≤ 0.05] reduced the PPTs at 0.75 h after injection in the contralateral hindpaw *c.f.* rats injected with the corresponding number of HK W256 cells, with a maximum reduction of 15.2 and 10.5% in the ipsilateral and the contralateral PPTs relative to the corresponding baseline PPTs, respectively (Supplementary Figure 6B).

##### Experiment 4

Administration of naloxone to group of rats given an ITI of DPBS or naïve control rats did not significantly alter PWTs in either the ipsilateral [*F*_(1,8,8/72)_= 0.2, 0.5, 0.9; *p* > 0.05] or the contralateral [*F*_(1,8,8/72)_ = 0.01, 0.1, 0.9; *p* > 0.05] PWTs (Supplementary Figure 6C). Similarly, naloxone did not significantly alter the ipsilateral [*F*_(1,8,8/72)_ = 0.3, 1.2, 0.9; *p* > 0.05] or contralateral [*F*_(1,8,8/72)_ = 1.3, 1.8, 0.5; *p* > 0.05] PPTs throughout the testing period in the same animals (Supplementary Figure 6D).

##### Experiment 5

**FIGURE 6:**
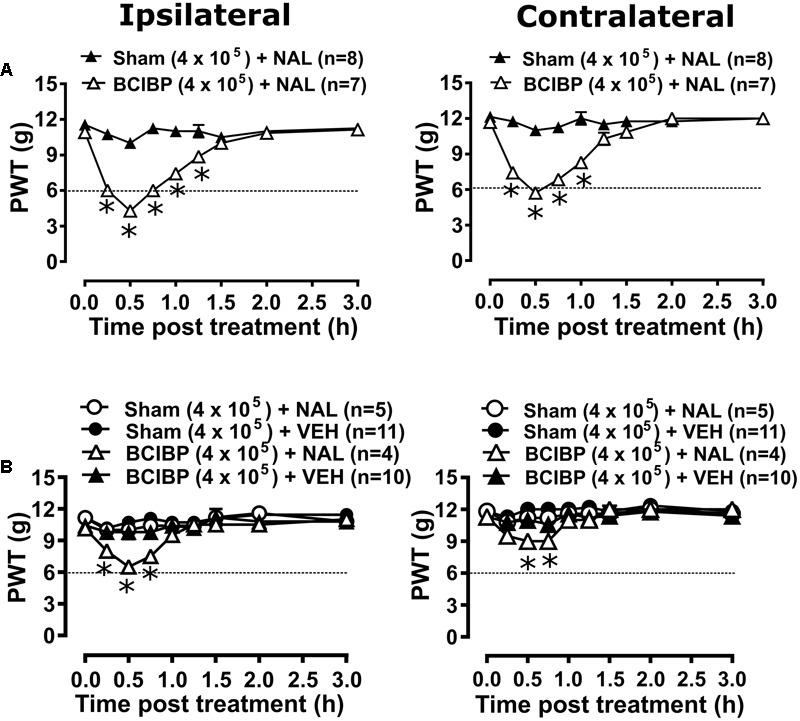
Effect of naloxone on ipsilateral and contralateral PWTs of rats. Panels in the figure show mean (±SEM) PWT versus time curves from experiment 5 following naloxone or vehicle injection between **(A)** day 43–51 post-ITI and **(B)** day 53–66 post-ITI. Dotted line indicates the threshold PWT value at/below which the rats were considered to have fully developed mechanical allodynia. BCIBP (4 × 10^5^), group of rats given an ITI of 4 × 10^5^ W256 cells; HK, heat-killed; NAL, naloxone (15 mg/kg s.c.); Sham (4 × 10^5^), group of rats given an ITI of 4 × 10^5^ HK W256 cells; VEH, vehicle; W256, Walker 256. ^∗^*p* ≤ 0.05 (Two-way ANOVA, *post hoc* Bonferroni test) *c.f.* rats given an ITI of HK W256 cells.

Administration of naloxone to group of rats administered a unilateral ITI of 4 × 10^5^ W256 cells in the time interval, day 43–51, post-ITI significantly [*F*_(1,8,8/104)_ = 173.7, 33.8, 22.5; *p* ≤ 0.05] reduced the PWTs from 0.25 to 1.25 h after naloxone injection in the ipsilateral hindpaw. Similarly, there was a significant [*F*_(1,8,8/104)_= 130.9, 27.5, 17.9; *p* ≤ 0.05] reduction in PWTs from 0.25 to 1 h after injection, in the contralateral hindpaws *c.f.* rats injected with 4 × 10^5^ HK W256 cells. The maximum decreases were 60.7 and 51.1% in the ipsilateral and contralateral PWTs relative to the corresponding baseline PWTs, respectively (**Figure 6A**). Administration of naloxone to these rats in the interval, day 53–66 post-ITI significantly [*F*_(3,8,24/208)_= 7.3, 9.8, 1.9; *p* ≤ 0.05] reduced the PWTs from 0.25 to 0.75 h in the ipsilateral hindpaws and significantly [*F*_(3,8,24/208)_= 6.5, 7.5, 1.8; *p* ≤ 0.05] reduced PWTs from 0.5 to 0.75 h in the contralateral hindpaws. The maximum PWT decreases were 35.8 and 20.4% in the ipsilateral and contralateral hindpaws relative to the corresponding baseline PWTs, respectively (**Figure 6B**). These findings were in contrast to the lack of effect observed in naloxone injected rats administered a unilateral ITI of 4 × 10^5^ HK W256 cells or vehicle injected rats administered a unilateral ITI of 4 × 10^5^ W256 cells and 4 × 10^5^ HK W256 cells.

##### Experiment 7

Administration of naloxone to rats administered a unilateral ITI of 4 × 10^5^ W256 cells did not significantly reduce the PTTs in either the ipsilateral [*F*_(3,8,24/112)_= 2.7, 0.9, 0.8; *p* > 0.05] or the contralateral [*F*_(3,8,24/112)_ = 0.2, 0.3, 1.1; *p* > 0.05] hindpaws throughout the testing (Supplementary Figure 6E).

The extent and duration of naloxone induced rescue of the pain phenotype in rats from these experiments are tabulated in Supplementary Table 3.

#### Anti-allodynic Effect of Morphine, Gabapentin, Amitriptyline and Meloxicam

**FIGURE 7:**
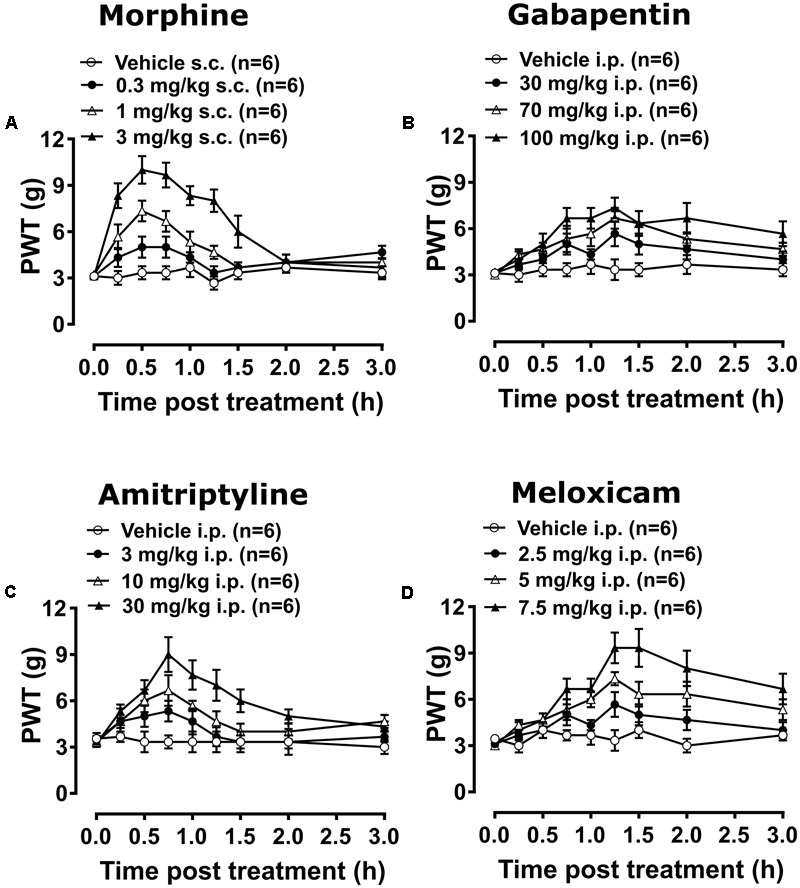
Temporal changes in PWTs of BCIBP rats in ipsilateral hindpaws following the administration of single bolus doses of analgesic and adjuvant drugs. Panels in the figure show temporal changes in mean (±SEM) PWT versus time curves following injection of **(A)** morphine, **(B)** gabapentin, **(C)** amitriptyline and **(D)** meloxicam.

Breast cancer-induced bone pain-rats having fully developed mechanical allodynia (PWTs ≤ 6g) in the ipsilateral hindpaws and administered single bolus doses of the clinically available analgesic drugs (morphine and meloxicam) or the adjuvant agents (gabapentin and amitriptyline), evoked unique pharmacological profiles consistent with their distinct modes of action. Anti-allodynia was produced in a dose-dependent manner by each of these drugs in both the ipsilateral (**Figures 7A–D**) and the contralateral hindpaws (Supplementary Figures 7A–D). Extent and duration of anti-allodynia (ΔPWT AUC) and ED_50s_ of each of the test compounds against mechanical allodynia in the ipsilateral and contralateral hindpaws, is summarized in **Table 2**.

**Table 2:** Extent and duration of anti-allodynia (ΔPWT AUC) and potencies of test compounds.

							ED_50_ for the alleviation of mechanical allodynia in the bilateral hindpaws (mg/kg)			
	Time (h)		Mean ΔPWT at peak effect (g)		ΔPWT AUC (g.h)		Ipsilateral		Contralateral	
Test compound and route of administration	Peak effect	_~_Duration of action	Ipsi	Contra	Ipsi	Contra	Mean	95% CI	Mean	95% CI
**Morphine (s.c.)**										
0.3 mg/kg	0.5–0.75	2	1.9 (±0.76)	2.2 (±1.07)	3.7 (±0.88)	3.9 (±1.28)	1.3	0.90–1.83	1.4	0.98–2.08
1 mg/kg			4.1 (±0.70)	4.2 (±0.72)	4.7 (±0.38)^∗^	4.8 (±0.49)^∗^				
3 mg/kg			6.9 (±1.00)	6.2 (±0.72)	9.6 (±1.22)^∗^	10.2 (±1.28)^∗^				
Vehicle			NA	NA	1.8 (±0.44)	2.2 (±0.38)				
**Gabapentin (i.p.)**										
30 mg/kg	1.25–1.5	>3	2.6 (±0.83)	2.4 (±0.78)	4.5 (±0.73)	4.5 (±0.61)	47.1	35.25–63.04	30.8	21.9–43.2
70 mg/kg			3.7 (±0.61)	3.7 (±0.68)	6.8 (±0.46)^∗^	7.5 (±0.63)^∗^				
100 mg/kg			4.1 (±0.70)	4.1 (±0.58)	8.4 (±1.08)^∗^	8.3 (±0.83)^∗^				
Vehicle			NA	NA	2.0 (±0.43)	3.0 (±0.76)				
**Amitriptyline (i.p.)**										
3 mg/kg	0.75–1.25	2	2 (±0.81)	2.1 (±0.78)	2.6 (±0.80)	2.6 (±0.75)	20.1	13.82–29.11	21.4	14.27–32.19
10 mg/kg			3.3 (±0.99)	2.8 (±0.78)	4.5 (±0.89)	4.0 (±0.99)				
30 mg/kg			5.8 (±1.15)	4.9 (±0.89)	7.8 (±1.28)^∗^	7.4 (±1.42)^∗^				
Vehicle			NA	NA	1.4 (±0.45)	1.7 (±0.27)				
**Meloxicam (i.p.)**										
2.5 mg/kg	1.25–1.5	>3	2.6 (±0.83)	2.2 (±0.66)	4.5 (±0.67)^∗^	4.5 (±0.65)^∗^	3.9	2.79–5.43	3.5	2.51–4.79
5 mg/kg			4.3 (±0.45)	4.3 (±0.45)	8.1 (±1.07)^∗^	8.2 (±1.00)^∗^				
7.5 mg/kg			6.1 (±0.96)	5.3 (±0.62)	11.5 (±1.22)^∗^	10.3 (±1.04)^∗^				
Vehicle			NA	NA	1.8 (±0.64)	2.0 (±0.68)				

*CI, confidence interval; Contra., contralateral hindpaws; Ipsi., ipsilateral hindpaws; NA, not applicable; ^∗^*p* ≤ 0.05 (One-way ANOVA, *post hoc* Dunnett’s multiple comparisons test) *c.f.* rats that received single bolus doses of vehicle.*

##### Experiment 10

Administration of 0.3 mg/kg morphine did not produce significant [*F*_(3,8,24/160)_ = 35.3, 18.1, 5.7; *p* > 0.05] anti-allodynia in the ipsilateral hindpaw throughout the testing, while it produced significant [*F*_(3,8,24/160)_ = 44.8, 17.6, 4.8; *p* ≤ 0.05] anti-allodynia between 0.5 and 0.75 h after injection in the contralateral hindpaw *c.f.* rats injected with vehicle. Administration of 1 mg/kg morphine produced significant [*F*_(3,8,24/160)_= 35.3, 18.1, 5.7; *p* ≤ 0.05] anti-allodynia between 0.25 and 1.25 h after injection in the ipsilateral hindpaw, while it produced significant [*F*_(3,8,24/160)_= 44.8, 17.6, 4.8; *p* ≤ 0.05] anti-allodynia between 0.25 and 1 h after injection in the contralateral hindpaw *c.f.* rats injected with vehicle. Administration of 3 mg/kg morphine produced significant anti-allodynia between 0.25 and 1.5 h after injection in both ipsilateral [*F*_(3,8,24/160)_= 35.3, 18.1, 5.7; *p* ≤ 0.05] and contralateral [*F*_(3,8,24/160)_= 44.8, 17.6, 4.8; *p* ≤ 0.05] hindpaws *c.f.* rats injected with vehicle.

##### Experiments 11 and 12

Gabapentin induced anti-allodynia was characterized by an onset of action which was delayed in nature. Administration of 30 mg/kg gabapentin produced significant [*F*_(3,8,24/160)_= 32.2, 7.9, 1.0; *p* ≤ 0.05] anti-allodynia in the ipsilateral hindpaw at 1.25 h after injection, while it did not produce significant [*F*_(3,8,24/160)_= 25.0, 7.0, 1.0; *p* > 0.05] anti-allodynia in the contralateral hindpaw throughout the testing *c.f.* rats injected with vehicle. Administration of 70 mg/kg gabapentin produced significant anti-allodynia between 1.25 and 1.5 h after injection in both ipsilateral [*F*_(3,8,24/160)_ = 32.2, 7.9, 1.0; *p* ≤ 0.05] and contralateral [*F*_(3,8,24/160)_= 25.0, 7.0, 1.0; *p* ≤ 0.05] hindpaws *c.f.* rats injected with vehicle. Administration of 100 mg/kg gabapentin produced significant [*F*_(3,8,24/160)_= 32.2, 7.9, 1.0; *p* ≤ 0.05] anti-allodynia in the ipsilateral hindpaw from 0.75 h to at least 3 h after injection, and it produced significant [*F*_(3,8,24/160)_= 25.0, 7.0, 1.0; *p* ≤ 0.05] anti-allodynia in the contralateral hindpaw from 1 h to at least 3 h after injection *c.f.* rats injected with vehicle. Administration of 3 mg/kg amitriptyline did not produce significant [*F*_(3,8,24/160)_= 15.3, 8.4, 2.0; *p* > 0.05] anti-allodynia in the ipsilateral hindpaw throughout the testing, whereas it produced significant [*F*_(3,8,24/160)_ = 17.7, 9.2, 2.9; *p* ≤ 0.05] anti-allodynia at 0.75 h after injection in the contralateral hindpaw *c.f.* rats injected with vehicle. Administration of 10 mg/kg amitriptyline produced significant anti-allodynia between 0.5 and 1 h after injection in both ipsilateral [*F*_(3,8,24/160)_= 15.3, 8.4, 2.0; *p* ≤ 0.05] and contralateral [*F*_(3,8,24/160)_= 17.7, 9.2, 2.9; *p* ≤ 0.05] hindpaws *c.f.* rats injected with vehicle. Administration of 30 mg/kg amitriptyline produced significant anti-allodynia between 0.5 and 1.5 h after injection in both ipsilateral [*F*_(3,8,24/160)_= 15.3, 8.4, 2.0; *p* ≤ 0.05] and contralateral [*F*_(3,8,24/160)_= 17.7, 9.2, 2.9; *p* ≤ 0.05] hindpaws *c.f.* rats injected with vehicle.

##### Experiment 13

Meloxicam induced anti-allodynia was characterized by an onset of action which was delayed in nature. Administration of 2.5 mg/kg meloxicam produced significant [*F*_(3,8,24/160)_= 54.1, 11.2, 2.3; *p* ≤ 0.05] anti-allodynia in the ipsilateral hindpaw at 1.25 h after injection, whereas it did not produce significant [*F*_(3,8,24/160)_= 50.6, 12.3, 2.0; *p* > 0.05] anti-allodynia in the contralateral hindpaw *c.f.* rats injected with vehicle. Administration of 5 mg/kg meloxicam produced significant anti-allodynia between 1 and 2 h after injection in both the ipsilateral [*F*_(3,8,24/160)_ = 54.1, 11.2, 2.3; *p* ≤ 0.05] and contralateral [*F*_(3,8,24/160)_= 50.6, 12.3, 2.0; *p* ≤ 0.05] hindpaws *c.f.* rats injected with vehicle. Administration of 7.5 mg/kg meloxicam produced significant anti-allodynia between 0.75 to at least 3 h after injection in both the ipsilateral [*F*_(3,8,24/160)_= 54.1, 11.2, 2.3; *p* ≤ 0.05] and contralateral [*F*_(3,8,24/160)_= 50.6, 12.3, 2.0; *p* ≤ 0.05] hindpaws *c.f.* rats injected with vehicle.

#### Anti-hyperalgesic Effect of Morphine, Gabapentin, Amitriptyline and Meloxicam

**FIGURE 8:**
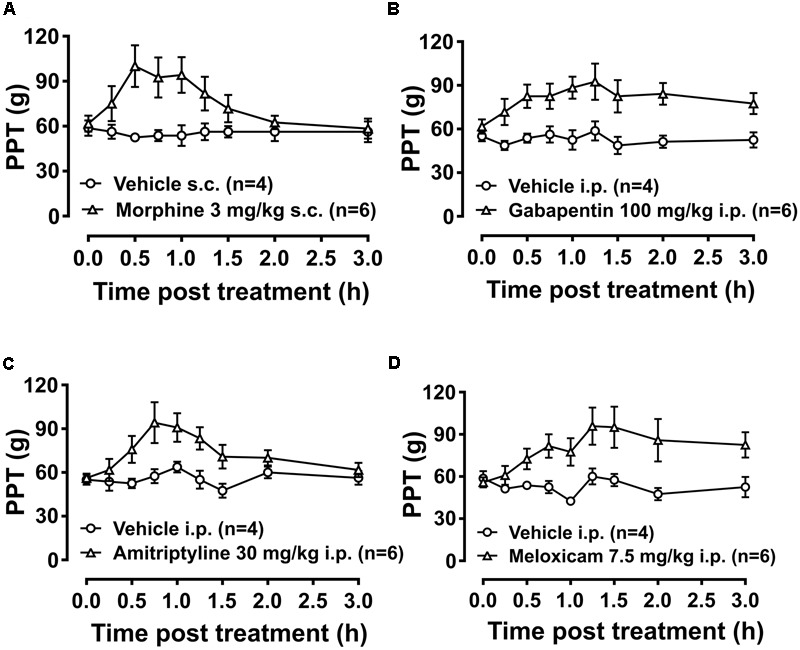
Temporal changes in PPTs of BCIBP rats in ipsilateral hindpaws following administration of the single bolus doses of analgesic and adjuvant drugs. Panels in the figure show temporal changes in mean (±SEM) PPT versus time curves following injection of **(A)** morphine, **(B)** gabapentin, **(C)** amitriptyline and **(D)** meloxicam.

Breast cancer-induced bone pain-rats having fully developed mechanical hyperalgesia (PPTs ≤ 80g) in the ipsilateral hindpaws and administered single bolus doses of the clinically available analgesic drugs (morphine and meloxicam) or the adjuvant agents (gabapentin and amitriptyline), evoked anti-hyperalgesia in both the ipsilateral (**Figures 8A–D**) and the contralateral hindpaws (Supplementary Figures 8A–D). Extent and duration of anti-hyperalgesia (ΔPPT AUC) of each of the test compounds in the ipsilateral and the contralateral hindpaws, is summarized in **Table 3**.

**Table 3:** Extent and duration of anti-hyperalgesia (ΔPPT AUC).

	Time (h)		Mean ΔPPT at peak effect (g)		ΔPPT AUC (g.h)	
Test compound and route of administration	Peak effect	~Duration of action	Ipsilateral	Contralateral	Ipsilateral	Contralateral
**Morphine (s.c.)**						
3 mg/kg	0.5	2	40 (±11.62)	36.7 (±10.88)	49.5 (±17.28)^∗^	46.7 (±17.21)^∗^
Vehicle			NA	NA	10.0 (±4.18)	9.2 (±4.85)
**Gabapentin (i.p.)**						
100 mg/kg	1.25	>3	32.5 (±10.7)	30.8 (±10.60)	64.2 (±19.79)	61.8 (±18.71)
Vehicle			NA	NA	9.7 (±3.48)	5.4 (±3.35)
**Amitriptyline (i.p.)**						
30 mg/kg	0.75	2	38.9 (±14.55)	36.7 (±13.42)	54.7 (±17.69)	53.3 (±17.30)
Vehicle			NA	NA	11.6 (±2.36)	10.5 (±3.69)
**Meloxicam (i.p.)**						
7.5 mg/kg	1.25–1.5	>3	41.7 (±14.64)	38.6 (±13.22)	85.0 (±33.62)	82.0 (±32.52)
Vehicle			NA	NA	8.1 (±4.69)	4.1 (±4.06)

*NA, not applicable; ^∗^*p* ≤ 0.05 (Mann–Whitney test) *c.f.* rats that received single bolus doses of vehicle.*

##### Experiment 14

Administration of 3 mg/kg morphine produced significant anti-hyperalgesia between 0.5 and 1 h in the ipsilateral hindpaw [*F*_(1,8,8/64)_= 5.5, 1.8, 2.7; *p* ≤ 0.05] and between 0.5 and 0.75 h in the contralateral hindpaw [*F*_(1,8,8/64)_= 5.1, 1.7, 2.4; *p* ≤ 0.05] *c.f.* rats injected with vehicle. Gabapentin induced anti-hyperalgesia was characterized by a delayed onset of action. Administration of 100 mg/kg gabapentin produced significant anti-hyperalgesia between 1 and 1.5 h in the ipsilateral hindpaw [*F*_(1,8,8/64)_= 9.8, 1.6, 1.2; *p* ≤ 0.05] and between 1 and 1.25 h in the contralateral hindpaw [*F*_(1,8,8/64)_= 11.6, 1.2, 1.7; *p* ≤ 0.05] *c.f.* rats injected with vehicle. Administration of 30 mg/kg amitriptyline produced significant relief of hyperalgesia at 0.75 h in both the ipsilateral [*F*_(1,8,8/64)_= 5.1, 3.9, 2.3; *p* ≤ 0.05] and contralateral [*F*_(1,8,8/64)_= 4.9, 3.9, 2.4; *p* ≤ 0.05] hindpaws *c.f.* rats injected with vehicle. Meloxicam induced anti-hyperalgesia was characterized by a delayed onset of action. Administration of 7.5 mg/kg meloxicam produced mild anti-hyperalgesia from 1.25 to at least 3 h in the ipsilateral hindpaws but this was not statistically significant; [*F*_(1,8,8/64)_= 5.9, 3.0, 2.6; *p* > 0.05]. By contrast, meloxicam at 7.5 mg/kg produced significant [*F*_(1,8,8/64)_ = 7.1, 2.7, 3.4; *p* ≤ 0.05] anti-hyperalgesia from 1.25 to at least until 3 h in the contralateral hindpaws *c.f.* rats injected with vehicle.

### Tibial Bone μCT Scan

#### Experiments 8 and 9

**FIGURE 9:**
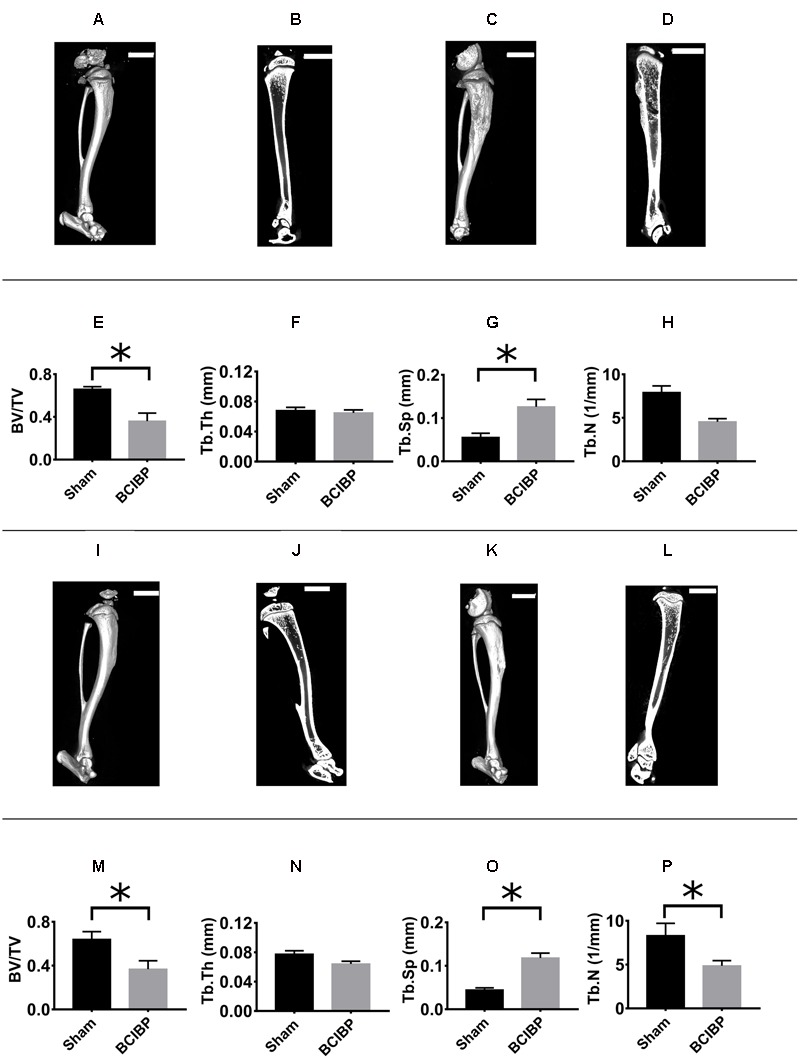
Radiological assessment of tibiae from BCIBP rats and corresponding sham rats. Panels in the figure show **(A)** 3D-μCT radiological image of a sham rat’s tibia at day 10 post-ITI, **(B)** trabecular bone of a sham rat’s tibia at day 10 post-ITI, **(C)** 3D-μCT radiological image of a BCIBP rat’s tibia at day 10 post-ITI, **(D)** trabecular bone of a BCIBP rat’s tibia at day 10 post-ITI, **(E–H)** morphometric changes in BCIBP rats’ tibiae relative to sham rats’ tibiae at day 10 post-ITI, **(I)** 3D-μCT radiological image of a sham rat’s tibia at day 48 post-ITI, **(J)** trabecular bone of a sham rat’s tibia at day 48 post-ITI, **(K)** 3D-μCT radiological image of a BCIBP rat’s tibia at day 48 post-ITI, **(L)** trabecular bone of a BCIBP rat’s tibia at day 48 post-ITI, **(M–P)** morphometric changes in BCIBP rats’ tibiae relative to sham rats’ tibiae at day 48 post-ITI. ^∗^*p* ≤ 0.05 (Two-way ANOVA, *post hoc* Bonferroni test). Scale bar – 5 mm.

The 3D-μCT radiological images and 2D-trabecular bone images of rats were obtained at day 10 post-ITI (**Figures 9A–D**) and day 48 post-ITI (**Figures 9I–L**).

##### BV/TV ratio

The BV/TV ratios for the proximal diaphyseal regions of tibiae from rats given an ITI of 4 × 10^5^ W256 cells and euthanized on day 10 (**Figure 9E**) [*F*_(1,1,1/4)_= 11.0, 0.03, 0.2; *p* ≤ 0.05] and day 48 (**Figure 9M**) [*F*_(1,1,1/4)_= 11.0, 0.03, 0.2; *p* ≤ 0.05] post-ITI, were significantly lower *c.f.* rats given an ITI of 4 × 10^5^ HK W256 cells and euthanized at the respective time points.

##### T_b_.T_h_

The T_b_.T_h_ of the proximal diaphyseal regions of tibiae from rats given an ITI of 4 × 10^5^ W256 cells and euthanized at day 10 (**Figure 9F**) [*F*_(1,1,1/4)_= 2.1, 167.9, 230.2; *p* > 0.05] or day 48 (**Figure 9N**) [*F*_(1,1,1/4)_ = 2.1, 167.9, 230.2; *p* > 0.05] post-ITI did not significantly change *c.f.* rats given an ITI of 4 × 10^5^ HK W256 cells and euthanized at the respective time points.

##### T_b_.S_p_

The T_b_.S_p_ of the proximal diaphyseal regions of tibiae of rats given an ITI of 4 × 10^5^ W256 cells and euthanized at day 10 (**Figure 9G**) [*F*_(1,1,1/4)_ = 21.1, 4.9, 0.1; *p* ≤ 0.05] and day 48 (**Figure 9O**) [*F*_(1,1,1/4)_= 21.1, 4.9, 0.1; *p* ≤ 0.05] post-ITI was significantly higher *c.f.* rats given an ITI of 4 × 10^5^ HK W256 cells and euthanized at the respective time points.

##### T_b_.N

The T_b_.N ratio of the proximal diaphyseal regions of tibiae of rats given an ITI of 4 × 10^5^ W256 cells and euthanized at day 48 (**Figure 9P**) post-ITI was significantly [*F*_(1,1,1/4)_= 9.0, 0.6, 0.007; *p* ≤ 0.05] lower than that for rats given an ITI of 4 × 10^5^ HK W256 cells on day 48 post-ITI. By contrast, for rats euthanized on day 10 (**Figure 9H**) post-ITI, the ratio was not significantly different [*F*_(1,1,1/4)_= 9.0, 0.6, 0.007; *p* > 0.05] from that of rats given an ITI of 4 × 10^5^ HK W256 cells and euthanized at day 10 post-ITI.

Significant decreases in the BV/TV ratio and in T_b_.N coupled with an increase in the T_b_.S_p_ were indicative of tumor induced osteolysis in the W256 cell injected tibiae.

### Tibial Bone Histology

#### Experiments 8 and 9

**FIGURE 10:**
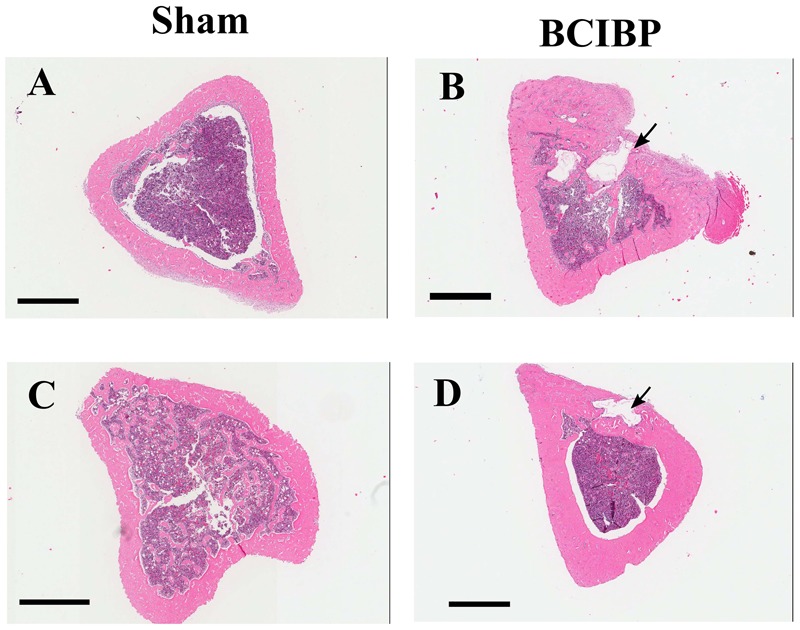
Histological assessment of tibiae from BCIBP rats and corresponding sham rats. Panels in the figure show representative images of H&E staining of tibial sections of **(A)** sham rat at day 10 post-ITI, **(B)** BCIBP rat at day 10 post-ITI, **(C)** sham rat at day 48 post-ITI and **(D)** BCIBP rat at day 48 post-ITI. Black arrowheads show destruction of cortical bone of tibiae. Scale bar – 1 mm.

Histological assessment of H&E stained sections showed marked osteolytic lesions due to bone destruction and immature *de novo* formation of bone in the proximal diaphyseal regions of the ipsilateral tibiae from groups of rats that had received a unilateral ITI of 4 × 10^5^ W256 cells at both day 10 (**Figure 10B**) and day 48 (**Figure 10D**) post-ITI. There were no such changes evident in the tibiae of sham-rats that had received a unilateral ITI of 4 × 10^5^ HK W256 cells and euthanized at the respective time points (**Figures 10A,C**).

### Immunocytochemistry of W256 Cells: Cytokeratin 18

**FIGURE 11:**
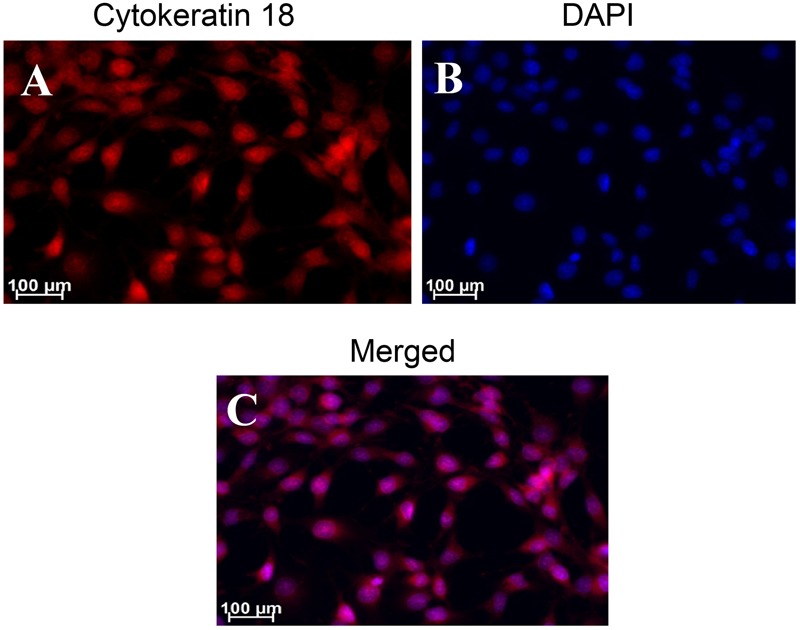
Immunocytochemical staining of Walker 256 cell line for Cytokeratin 18 using ab187573 (Abcam) antibody. Panels in the figure show **(A)** cytokeratin 18 **(B)** DAPI and **(C)** A and B merged.

Using the anti-Cytokeratin 18 antibody [C04] (Alexa Fluor^®^ 488) ab187573 (Abcam), cultured and fixed W256 cells showed immunofluorescent staining (**Figure 11**) which was in accord with Cytokeratin 18 expression in ATCC W256 cells reported by others (Lewis et al., 2013). Similar results were obtained with anti-Cytokeratin 18 antibody [C-04] ab668 (Abcam) which was used as a second confirmatory antibody (Supplementary Figure 9).

### Tibial Bone Immunohistochemistry

#### Experiment 15 and 16

**FIGURE 12:**
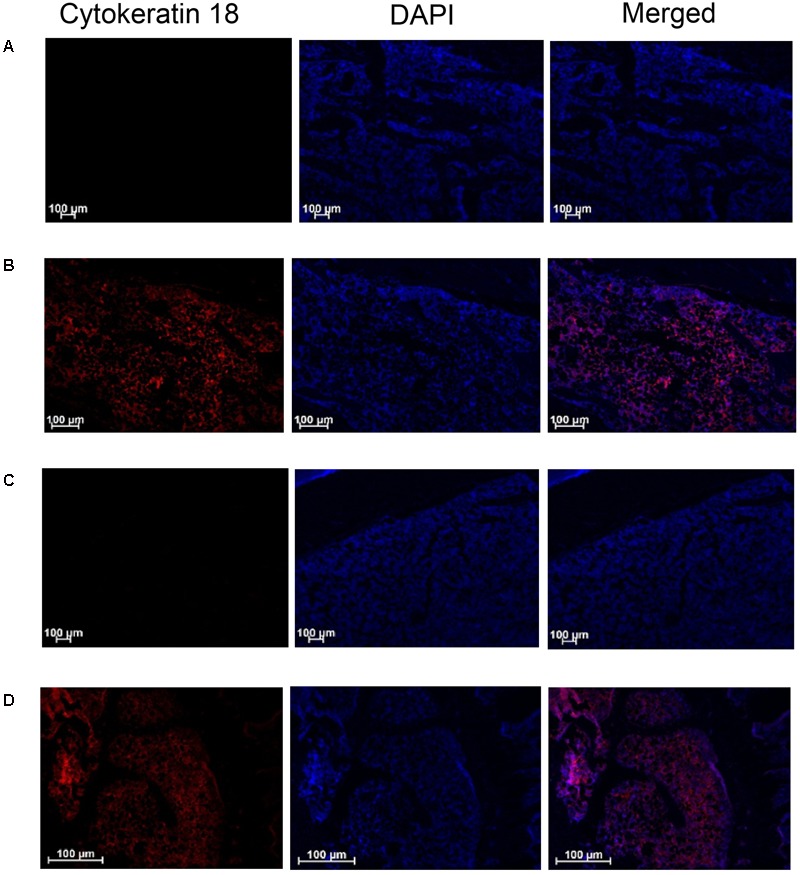
Immunohistochemical staining of Cytokeratin 18 in tibial sections of BCIBP rats and corresponding sham rats using ab187573 (Abcam) antibody. Panels in the figure show immunofluorescence imaging of tibial sections of **(A)** sham rat at day 7 post-ITI, **(B)** BCIBP rat at day 7 post-ITI, **(C)** sham rat at day 38 post-ITI and **(D)** BCIBP rat at day 38 post-ITI.

The presence of W256 cancer cells in longitudinal sections of the ipsilateral tibiae from rats given these cells by unilateral ITI was confirmed by immunohistochemical staining using the anti-Cytokeratin 18 antibody [C04] (Alexa Fluor^®^ 488) ab187573 (Abcam). Cytokeratin 18 immunofluorescence was observed in sections from BCIBP-rats’ tibiae collected on both day 7 (**Figure 12B**) and day 38 (**Figure 12D**) after unilateral ITI of 4 × 10^5^ W256 cells. Importantly, in the corresponding tibial sections from sham-rats administered a unilateral ITI of 4 × 10^5^ HK W256 cells, specific immunofluorescence for Cytokeratin 18 was absent at both time points (**Figures 12A,C**). Similar results were obtained with another anti-Cytokeratin 18 antibody ([C-04] ab668 (Abcam)) in the experiment that was used as a confirmatory step (Supplementary Figure 10).

## Discussion

In the present study, we have established an optimized model of BCIBP in rats successfully and have comprehensively characterized the model using behavioral, radiological, pharmacological, histological, and immunohistochemical methods. Our optimized model successfully addresses the issue of poor general animal health that is a major limitation of previously described models. Using female Wistar Han rats, we are the first to show that the severity (magnitude/extent) of Walker 256 cell-induced mechanical allodynia and mechanical hyperalgesia, is directly correlated with the initial number of cancer cells inoculated in the tibiae. Following unilateral ITI of W256 cells, rats in the present study developed bilateral mechanical allodynia and mechanical hyperalgesia, which is the most common manifestation in CIBP (Paley et al., 2011). Cancer-induced bone destruction was observed in the tibiae of rats using both radiological and histological assessments, in concordance with osteolytic changes seen in patients with breast cancer metastases to the skeleton (O’Sullivan et al., 2015; Woolf et al., 2015). The model was also found to be suitable for screening of analgesic drugs with diverse mechanisms of action.

Animal models involving injection of cancer cells in bone are commonly used in experimental research to study pathophysiological mechanisms of pain hypersensitivities and disease progression (Kaan et al., 2010; Graham et al., 2014; Taipaleenmäki et al., 2015; Liu et al., 2016; Torrano et al., 2016). Using mice and rats of different ages, weight ranges, sexes, strains and various breeding and housing conditions, CIBP studies employ a wide variety of cultured cancer cell lines including lung cancer (e.g., Lewis Lung), prostate cancer (e.g., PC3N, ACE-1, AT-3), breast cancer (e.g., 66.1, 4T1, MDA-MB-231, MDA-MB-231-TXSA, MRMT1), colon cancer (e.g., Colon-26), osteosarcoma (NCTC 2472) and fibrosarcoma (e.g., MC57G) (Pacharinsak and Beitz, 2008; Currie et al., 2013, 2014; Lozano-Ondoua et al., 2013; Falk and Dickenson, 2014; Slosky et al., 2015). These cell lines which are injected into the tibia, humerus, femur and calcaneus in various CIBP studies (Falk and Dickenson, 2014) can give rise to distinct pain behaviors and neurochemical changes (Sabino et al., 2003). The features of any given animal model of CIBP thus require characterisation using standardized protocols to reduce the number of variables affecting the experiments to enhance experimental reproducibility. In the present work, W256 cells were selected as they are widely used in research (Justice, 1985; Brigatte et al., 2007) and because of the ease with which they can be cultured, standardized and reproduced (McEuen and Thomson, 1933; Hang et al., 2015). A comparative summary of previous studies using the W256 cell-induced bone pain model in rats, has been comprehensively provided in a recent review (Shenoy et al., 2016).

Until recently, the animal models of CIBP involved injection of cancer cells systemically, which resulted in deterioration of the overall animal health due to metastases to vital organs like the lungs, liver, brain and throughout the skeleton (Urch, 2004; Simmons et al., 2015). In CIBP models based upon the ITI of cancer cells, distant metastases can occur due to the cells escaping from the drilled hole or due to osteolysis of the bones, leading to severe degradation of the animal health and loss of body weight (Muralidharan et al., 2013). Hence, it is very important that all of the animals involved in the CIBP studies are closely monitored throughout the experimental period. Our work herein is the first to systematically assess and document clinical health parameters in animals following ITI of W256 cells. We carefully monitored all animals and found that the number with deteriorated health was very low. Such animals were immediately excluded from the experiments and were humanely euthanized for ethical reasons.

Interestingly, mechanical hypersensitivity also developed in the contralateral hindpaws of rats given a unilateral ITI of W256 cells, although it was less intense than that evoked in the ipsilateral hindpaws. We are the first to show that the number of W256 cells injected in the tibiae is a predisposing factor influencing the nature of pain hypersensitivities developed. With a lower number of cells inoculated, the pain hypersensitivities developed are unilateral in nature (developed in ipsilateral hindpaws). By increasing the number of cells, pain hypersensitivities become bilateral in nature (in both ipsilateral and contralateral hindpaws). Peripherally acting mechanisms like the involvement of circulating factors and transmedian sprouting, or centrally acting mechanisms like signaling through the commissural interneurons of the spinal cord could be the cause of contralateral mirror effects associated with unilateral injury (Koltzenburg et al., 1999). These contralateral effects could also be associated with activation of spinal glial cells and proinflammatory cytokine secretion, as well as morphological changes in the local tibial nerves, similar to effects that have been reported in a model of sciatic inflammatory neuritis (Chacur et al., 2001). Inoculation of breast cancer cells in bones is known to produce massive sprouting of sensory nerve fibers of the periosteum in rodent models of CIBP (Bloom et al., 2011). Noxious inputs persistently coming from the periphery can sensitize some areas of the brain, thereby affecting the pain circuitry causing bilateral hypersensitivity via the descending pain control system (Ikeda et al., 2007; Meeus and Nijs, 2007). Thermal hyperalgesia did not develop in rats given a unilateral ITI of W256 cells. The absence of thermal hyperalgesia is in contrast to pain behaviors observed after ITI of AT3B prostate cancer cells (Muralidharan et al., 2013), suggesting that tumor-specific interactions with the bone environment and/or peripheral nerves innervating the bone contribute to this difference. Thermal and mechanical pain behaviors are known to be underpinned by diverse mechanisms (Paqueron et al., 2003; Wang et al., 2012). Nociceptors present in the skin are sensitized by thermal stimuli, while the nociceptors which are deeply situated in the somatic tissues like joints and muscle are highly sensitive to mechanical stimuli (Schaible, 2007).

After approximately 3 weeks post-surgery, the mechanical allodynia and mechanical hyperalgesia in the hindpaws of rats administered a unilateral ITI of W256 cells spontaneously resolved and this state was maintained until the end of the study. While all the existing studies using this model typically assess the pain behaviors only until around 20–25 days after injection of cancer cells, our findings are the first to successfully investigate the model for up to 66 days and to show that W256 cell-induced bone pain can apparently resolve spontaneously at later stages despite the ongoing presence of cancer cells, similar to the clinical situation in humans. The beginning of resolution of pain hypersensitivity behavior around 20–25 days after surgery in this model has been observed in other studies, although these studies only assessed pain behaviors until 20–25 days (Zhao et al., 2010; Xu et al., 2013; Huang et al., 2014). A similar spontaneous reversal of mechanical hypersensitivity has been reported in rats administered a unilateral ITI of MRMT-1 cells in a BCIBP model (Falk et al., 2015). Cancer metastases to bones without clinical pain or other symptoms is not uncommon (Costelloe et al., 2009; Phanphaisarn et al., 2016). In a recent retrospective patient record study involving 1105 women with breast cancer skeletal metastases, it was found that the burden of symptoms including pain decreased after the diagnosis of bone metastases, although there was no substantial increase in use of analgesic medication after the diagnosis of bone metastases compared to the scenario before the diagnosis (Cleeland et al., 2016). In this study, only a small proportion of patients (~16%) were newly introduced to analgesic medications or had the dose of their existing medications increased after the diagnosis of bone metastases. These observations are consistent with the notion that the human body has endogenous pain-relieving mechanisms in place to attenuate pain due to progression of metastatic disease and so limit the presentation of symptoms.

The work presented herein is the first to use histology, radiology and immunohistochemistry to provide evidence that the cancer disease remains persistent in the rat tibiae, despite apparent resolution of pain hypersensitivities at later stages of this model. We chose immunohistochemistry as a technique to stain tibial sections, because it provides direct evidence of the presence of cancer cells (Slade and Coombes, 2007). The presence of cancer cells in the tibiae during both the pain state and the resolved-pain state was confirmed with immunohistochemical staining of Cytokeratin 18. Endogenous opioids potentially play a very important role in cancer pain attenuation (Roques et al., 2012; Lesniak et al., 2016; Zhao et al., 2016). We administered naloxone, which is a non-selective antagonist of opioid receptors (van Dorp et al., 2007), to assess whether similar mechanisms might contribute in the present model and we are the first to show that naloxone rescued the pain phenotype. The observed re-emergence of pain behaviors after naloxone administration not only further supports the continued presence of tumor cells in bone, but also supports the hypothesis that the endogenous opioid system could be at least partially responsible for the spontaneous resolution of pain hypersensitivity. A CIBP model involving an ITI of prostate cancer cells in rats showed similar spontaneous resolution of pain due to up-regulation of the endogenous opioid system (Muralidharan et al., 2013). Studies in mice using an osteosarcoma-induced bone pain model also showed such a spontaneous resolution of hypersensitivity in the hindpaws that was unmasked by administration of naloxone to reverse the analgesia produced by up-regulated endogenous opioids (Menendez et al., 2003; Baamonde et al., 2006). Similarly, several other studies have found that the endogenous opioid system might play a key role in resolution of cancer pain hypersensitivities (Sevcik et al., 2006; Menendez et al., 2008). While upregulation of endogenous opioids has been implicated in the resolution of cancer pain in a number of studies (Li et al., 2016), involvement of other complex neuroimmune mechanisms is also possible. For instance, lipoxins and endogenous lipoxygenase-derived eicosanoids – members of the lipid mediators class with a wide spectrum of effects against inflammation and nociception– suppress the spinal expression of pro-inflammatory cytokines and might also be partially responsible for spontaneous resolution of W256 cell-CIBP in this model (Hu et al., 2012).

While the continued presence of tumor load was confirmed by immunohistochemical analysis at a state when the pain hypersensitivity was resolved in this model, elimination of cancer cells could also contribute to the observed resolution of pain behaviors. The phenomenon of breast cancer regression is well-known in humans (Lewison, 1976; Hutter, 1982; Burnside et al., 2006; Barry, 2009; Onuigbo, 2012). Similarly, W256 breast cancer cells also have the potential to transform into a variant known for its regression *in vivo* (Guimarães et al., 2010), causing cancer regression with the prolonged duration of the study (Jensen and Muntzing, 1970; Cavalcanti et al., 2003; Schanoski et al., 2004).

Our present work is the first to pharmacologically characterize the W256 cell-induced bone pain model in rats and to show that the model is responsive to clinically used analgesic drugs that have different mechanisms of action. Morphine, gabapentin, amitriptyline and meloxicam are important drugs representing diverse analgesic drug classes used in treating cancer pain (Mantyh et al., 2002). Morphine, an agonist predominantly at the μ-opioid (MOP) receptor, is one of the most important members of the strong opioid analgesic class (Cao et al., 2010). All three major opioid receptor types (μ, δ, κ) belong to the superfamily of *G*_i_/*G*_o_-protein-coupled receptors (McDonald and Lambert, 2005). Opioid agonists enhance the opening of G-protein-activated inwardly rectifying K^+^ (GIRK) channels (Ikeda et al., 2000); inhibit the opening of voltage-gated calcium channels, and inhibit cAMP production by adenylate cyclase. The net effect is inhibition of nociception (Rang et al., 2011). The ED_50-Ipsilateral_ of morphine in this model (1.3 mg/kg) was found to be higher than that in a cisplatin induced peripheral neuropathy model (0.8 mg/kg; s.c.) (Han et al., 2014), similar to that in a neuropathic pain model of spared nerve injury (1.2 mg/kg; s.c.) (Zhao et al., 2004), but lower than that in nociceptive paw pressure test (2.8 mg/kg; s.c.) (Morgan et al., 2006) in rats. Our findings are aligned with others who showed that morphine is effective in alleviating pain hypersensitivities in this model (Guo et al., 2017; Liu et al., 2017). In other work, spinal levels of endomorphin-2, an endogenous ligand at μ-opioid receptors, were reduced following ITI of W256 cells, and this was correlated with development of mechanical allodynia in these rats (Chen et al., 2015). Similarly, mRNA and protein levels of μ-opioid receptors in the spinal dorsal horn and dorsal root ganglia of rats were reduced following unilateral ITI of W256 cells in this model (Yao et al., 2016; Hou et al., 2017). Gabapentin is used in both epilepsy and neuropathic pain. It inhibits calcium currents via high-voltage-activated channels containing the α_2_δ-1 subunit, which in turn decreases the release of neurotransmitters and reduces the excitability postsynaptically. Other mechanisms have also been proposed including modest actions on voltage gated potassium channels (Sills, 2006). The ED_50-Ipsilateral_ of gabapentin in this model (47.1 mg/kg) was higher than that reported in a neuropathic pain model of spinal nerve ligation (34 mg/kg; i.p.) in rats (Hunter et al., 1997). Tricyclic antidepressant drugs such as amitriptyline are also used as analgesic adjuvant drugs, which augment descending noradrenergic inhibition and are utilized for the management of neuropathic pain conditions (Tura and Tura, 1990). NSAIDs such as meloxicam inhibit COX, the enzyme responsible for the conversion of arachidonic acid into prostaglandin H_2_, which is an initial step in the synthesis of PGE2, a mediator of inflammation. At therapeutic doses, meloxicam has modest selectivity for inhibition of COX-2 over COX-1 (Noble and Balfour, 1996). The ED_50-Ipsilateral_ of morphine and gabapentin in our optimized model (1.3 and 47.1 mg/kg, respectively) were lower than that reported in prostate CIBP model (1.9 and 78.0 mg/kg, respectively), whereas the ED_50-Ipsilateral_ of amitriptyline and meloxicam (20.1 and 3.9 mg/kg, respectively) were higher than that determined in the prostate CIBP model (14.9 and 2.6 mg/kg, respectively) in rats (Muralidharan et al., 2013). Morphine, gabapentin, amitriptyline and meloxicam produced dose-dependent analgesia in our optimized rat model of BCIBP, with each of the drugs having a unique pharmacological profile in terms of extent and duration of action and time of peak effect. Considering the ED_50_ doses (mg/kg), the potency rank order was: morphine > meloxicam > amitriptyline > gabapentin. Hence, our model was found to be suitable for assessing the pain-relieving effects of compounds from diverse pharmacological drug classes that are used clinically to alleviate CIBP.

Pain is one of the major problems in cancer patients having metastases to their bones (Tsuzuki et al., 2016). CIBP is a complex pathological manifestation of inflammatory, neuropathic and cancer-specific or tumorigenic components (Cao et al., 2010). Efficient preclinical models of CIBP are essential to understand the underlying complexities and to assist in the drug discovery processes aimed at relieving the debilitating pain (Blouin et al., 2005). In the present work, we systematically injected a number of different concentrations of W256 cells in a rat model and assessed the health characteristics of the animals. The development of pain hypersensitivity depended upon the initial number of cells inoculated, while excellent animal health was maintained at all cell numbers assessed. Analogous to clinical practice, we used radiological and histological techniques to evaluate cancer-induced bone changes (Mertens et al., 1998; Heck et al., 2006; Menezes and Ahmed, 2014) and found profound osteolysis. This observation is in contrast to prostate cancer metastases to bones, in which mixed osteolytic-osteogenic bone lesions are formed with osteosclerotic effects predominating in both the clinic and the animal models (Ibrahim et al., 2010; Muralidharan and Smith, 2013). Although the cancer cells were still present in the tibiae of animals herein, the hindpaw hypersensitivity of rats resolved at later stages of the model and the endogenous opioid system was at least partially responsible for this spontaneous pain resolution. We performed detailed pharmacological profiling using standard analgesic drugs used to treat CIBP in the clinical setting and found that this model could be useful to assess novel analgesic compounds with diverse mode of actions.

## Author Contributions

All authors (PS, AK, IV, and MS) meet the essential authorship criteria required by the journal to be observed including (a) substantial contributions to the conception or design of the work; or the acquisition, analysis, or interpretation of data for the work; and (b) drafting the work or revising it critically for important intellectual content; and (c) final approval of the version to be published; and (d) agreement to be accountable for all aspects of the work in ensuring that questions related to the accuracy or integrity of any part of the work are appropriately investigated and resolved.

## Conflict of Interest Statement

The authors declare that the research was conducted in the absence of any commercial or financial relationships that could be construed as a potential conflict of interest.

## Abbreviations

AUC: area under curve
BCIBP: breast cancer-induced bone pain
CIBP: cancer-induced bone pain
HK: heat-killed
ITI: intra-tibial injection
PPT: paw pressure threshold
PTT: paw thermal threshold
PWT: paw withdrawal threshold
W256: Walker 256 cells
