# Supplementary material for: Optimization and In Vivo Profiling of a Refined Rat Model of Walker 256 Breast Cancer Cell-Induced Bone Pain Using Behavioral, Radiological, Histological, Immunohistochemical and Pharmacological Methods
Source: Front Pharmacol. 2017 Jul 6;8:442. doi: 10.3389/fphar.2017.00442 (PMC5498471; doi:10.3389/fphar.2017.00442)
Supplement: Supplementary file 1 [file Data_Sheet_1.pdf]

Supplementary Material

Optimization and *In Vivo* Profiling of a Refined Rat Model of Walker 256 Breast Cancer Cell-Induced Bone Pain Using Behavioral, Radiological, Histological, Immunohistochemical and Pharmacological Methods

Priyank Shenoy[1, 2], Andy Kuo[1], Irina Vetter[3,4], Maree T. Smith[1,4*]

[1]Centre for Integrated Preclinical Drug Development, Centre for Clinical Research, The University of Queensland, Brisbane, QLD, Australia

[2]School of Biomedical Sciences, The University of Queensland, Brisbane, QLD, Australia

[3]Institute for Molecular Bioscience, The University of Queensland, Brisbane, QLD, Australia

[4]School of Pharmacy, The University of Queensland, Brisbane, QLD, Australia

***Correspondence:**
Professor Maree T. Smith
Email: maree.smith@uq.edu.au

Address:
Centre for Integrated Preclinical Drug Development (CIPDD), Level 3, Steele Building,
Centre for Clinical Research (UQCCR)
Faculty of Medicine
St Lucia Campus
The University of Queensland
Brisbane, QLD 4072
Australia.

Tel: +61-7-33652554
Fax: +61-7-33467391

1 Supplementary Tables

Supplementary Table 1. Mean (±SEM) values of behavioural tests of rats given an ITI of HK cells as a control for lack of pain hypersensitivity.

Experiment no.	Day post-ITI	Mean PWT (g)		Mean PPT (g)		Mean PTT (sec)	
		Ipsi	Contra	Ipsi	Contra	Ipsi	Contra
7	21	-	-	-	-	13.3 (± 0.25)	13.5 (± 0.49)
10	8<br>13	10<br>11.3	11.3<br>12	-	-	-	-
11	8<br>10<br>15	10.7<br>10.7<br>10	11.3<br>10.7<br>10.7	-	-	-	-
12	7<br>9<br>11<br>14	11.3<br>11.3<br>12<br>12	12<br>10.7<br>12.7<br>12.7	-	-	-	-
13	7<br>9<br>12	10.7<br>11.3<br>10.7	12<br>12<br>12	-	-	-	-
14	7<br>10<br>12<br>14	-	-	115<br>111.7<br>116.7<br>110	125<br>116.7<br>123.3<br>116.7	-	-

Contra, contralateral hindpaws; Ipsi, ipsilateral hindpaws; -, not assessed

Supplementary Table 2. Mean (±SEM) values of baseline behavioural tests of rats prior to surgeries / ITI.

**Experiment no.**	**Mean pre-surgery baseline PWT (g)**				**Behavioural test value; unit**
	**W256 cells' group**		**HK cells' group**		
	**Ipsi**	**Contra**	**Ipsi**	**Contra**	
7	12.9 (± 0.65)	13.1 (± 0.79)	10.8 (± 0.51)	11.7 (± 0.16)	PTT; sec
10	10.1 (± 0.28)	10.5 (± 0.24)	9.3	10	PWT; g
11	10.2 (± 0.25)	10.7 (± 0.23)	9.3	10	PWT; g
12	10.6 (± 0.31)	10.7 (± 0.40)	10.7	11.3	PWT; g
13	10.5 (± 0.37)	10.3 (± 0.38)	11.3	10.7	PWT; g
14	102.1 (± 2.41)	104.8 (± 2.55)	105	108.3	PPT; g

Contra, contralateral hindpaws; Ipsi, ipsilateral hindpaws

Supplementary Table 3. Extent and duration of naloxone induced rescue of pain phenotype.

Exp. No.	Days post ITI	ITI	Drug treatment	ΔPWT AUC (g.h)		ΔPPT AUC (g.h)		ΔPTT AUC (sec.h)		Time (h)	
				Ipsi	Contra	Ipsi	Contra	Ipsi	Contra	Peak effect	~Duration of action
3	82-92	1.5 x 10^5 W256<br>1.5 x 10^5 HK W256	Naloxone<br>Naloxone	-<br>-	-<br>-	22.2 (± 4.76)<br>9.3 (± 4.44)	14.8 (± 3.91)<br>4.2 (± 2.13)	-<br>-	-<br>-	0.75<br>NE	1<br>NE
3	81-91	1.5 x 10^5 W256<br>1.5 x 10^5 HK W256	Naloxone<br>Naloxone	4.1 (± 1.18)<br>1.8 (± 0.50)	4.4 (± 1.13)<br>1.5 (± 0.25)	-<br>-	-<br>-	-<br>-	-<br>-	0.5<br>NE	1<br>NE
4	82-94	DPBS<br>Naïve	Naloxone<br>Naloxone	-<br>-	-<br>-	7.2 (± 2.30)<br>5.3 (± 1.75)	10.7 (± 5.75)<br>13.2 (± 5.41)	-<br>-	-<br>-	NE<br>NE	NE<br>NE
4	81-93	DPBS<br>Naïve	Naloxone<br>Naloxone	0.9 (± 0.35)<br>0.9 (± 0.56)	0.7 (± 0.33)<br>1.3 (± 0.72)	-<br>-	-<br>-	-<br>-	-<br>-	NE<br>NE	NE<br>NE
5	43-51	4 x 10^5 W256	Naloxone	6.2 (± 0.75)*	5.5 (± 0.70)*	-	-	-	-	0.5	1.5

		4 x 10^5 HK W256	Naloxone	2.5 (± 0.55)	1.7 (± 0.39)	-	-	-	-	NE	NE
5	53-66	4 x 10^5 W256	Naloxone	2.5 (± 0.35)	1.8 (± 0.57)	-	-	-	-	0.5-0.75	1.25
		4 x 10^5 W256	Vehicle	1.1 (± 0.74)	1.1 (± 0.50)	-	-	-	-	NE	NE
		4 x 10^5 HK W256	Naloxone	1.9 (± 0.38)	1.6 (± 0.44)	-	-	-	-	NE	NE
		4 x 10^5 HK W256	Vehicle	1.7 (± 0.48)	0.6 (± 0.18)	-	-	-	-	NE	NE
7	21-24	4 x 10^5 W256	Naloxone	-	-	-	-	2.2 (± 1.06)<br>3.1 (± 0.77)	3.3 (± 1.06)	NE	NE
		4 x 10^5 W256	Vehicle	-	-	-	-	2.1 (± 1.33)	1.4 (± 0.59)	NE	NE
		4 x 10^5 HK W256	Naloxone	-	-	-	-	0.7 (± 0.33)	2.1 (± 1.31)	NE	NE
		4 x 10^5 HK W256	Vehicle	-	-	-	-		0.9 (± 0.16)	NE	NE

Contra, contralateral hindpaws; Ipsi, ipsilateral hindpaws; NE, no drug effect; -, not assessed. *$p \leq 0.05$ (Unpaired t test) *c.f.* rats administered a unilateral ITI of 4 x 10^5 HK W256 cells that received single bolus dose of naloxone

2 Supplementary Figures

THE UNIVERSITY OF QUEENSLAND CIPDD

CD-FM-AN30 Clinical Observation Record

Study No.:____________ **Animal Chip No.:**_____________ **Cage No.:** _____ **Week:**_________

**Observation**	**Date/Study Day**						
Operator							
Animal Weight (g)							
Mortality							
Moribund							
No abnormalities detected (NAD) (animal is well and healthy)	❐	❐	❐	❐	❐	❐	❐
**Grading of Observations: M = Mild; Mo = Moderate; S = Severe**							
**Skin and Fur**							
piloerection							
lack of grooming							
fur loss							
irritation or sores							
**Eyes & mucus membranes**							
palpebral closure							
eyes cloudy							
ocular or nasal discharge (including chromodacryorrhea)							
excessive salivation							
diarrhoea							
bleeding from orifice							
**Respiratory & circulatory function**							
increased respiratory rate							
decreased respiratory rate							
gasping							
blueness of extremities							
**Gait & posture**							
hunched posture or gait							
ataxia							
excessive sway, rocks or lurches							
hind limbs show exaggerated or overcompensated movements, drag or are splayed							
feet point markedly outward from body							
forelimbs drag, are extended or unable to support weight							
walks on tiptoe							
lameness							
**Behaviour**							
reduced activity levels							
increased activity levels							
circling							
stereotypic grooming							
pacing							
repetitive sniffing							
head weaving							
writhing or flopping							
retropulsion							
straub tail							
self-mutilation / autotomy							
**Clonic Tremors or Convulsions**							
repetitive movements of mouth and jaws							
quivers of limbs, ears, head or skin							
tremors (mild, moderate or severe)							
myoclonic jerks							
clonic convulsions							
wet dog shakes							
**Tonic Tremors or Convulsions**							
contraction of extensors							
head and body rigidly arched							
jumps with all feed leaving surface							
severe clonic and /or tonic convulsions resulting in dyspnoea, postictal depression or death							
**Comments**							

Any of the following unexpected observations noted below will be considered as potential humane endpoints for the animal, whereby staff and students must notify the CI/Study Director and the OIC immediately. In the event that the CI/Study Director or the OIC is not immediately contactable, staff and students will euthanize the animal.

(1) Animal is moribund (2) Weight loss > 10% overnight or >20% over 3 consecutive days (3) Two or more "severe" scores are noted in the Clinical Observations

CD-FM-AN30 Clinical Observation Record
Page 1 of 1

Supplementary Figure 1. Form used to record clinical observations in rats.

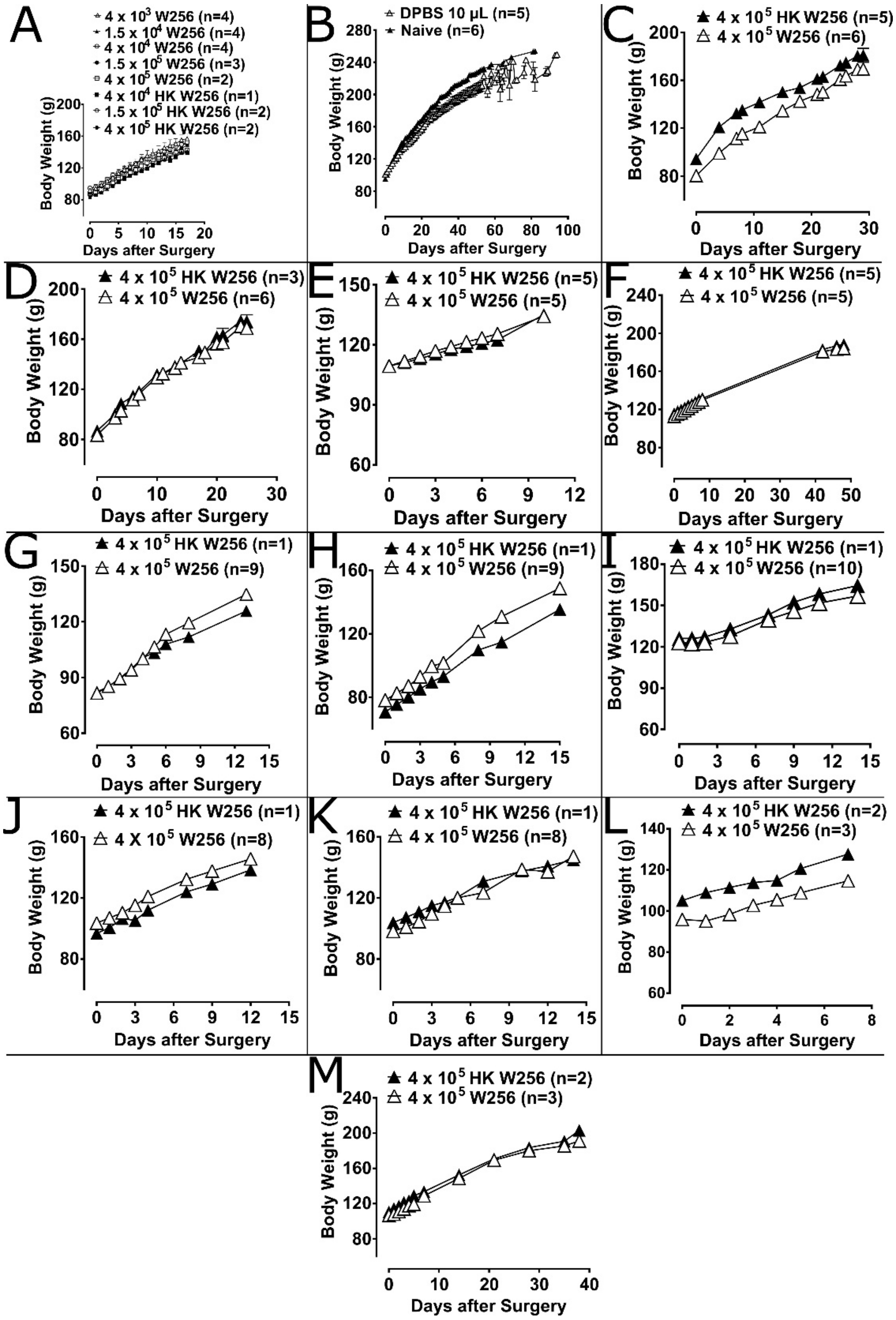

Supplementary Figure 2. Body weight of rats from various experiments. Panels in the figure show mean (±SEM) body weight of rats from (A) experiment 1, (B) experiment 4, (C) experiment 6, (D) experiment 7, (E) experiment 8, (F) experiment 9, (G) experiment 10, (H) experiment 11, (I) experiment 12, (J) experiment 13, (K) experiment 14, (L) experiment 15 and (M) experiment 16. HK, heat-killed; W256, Walker 256.

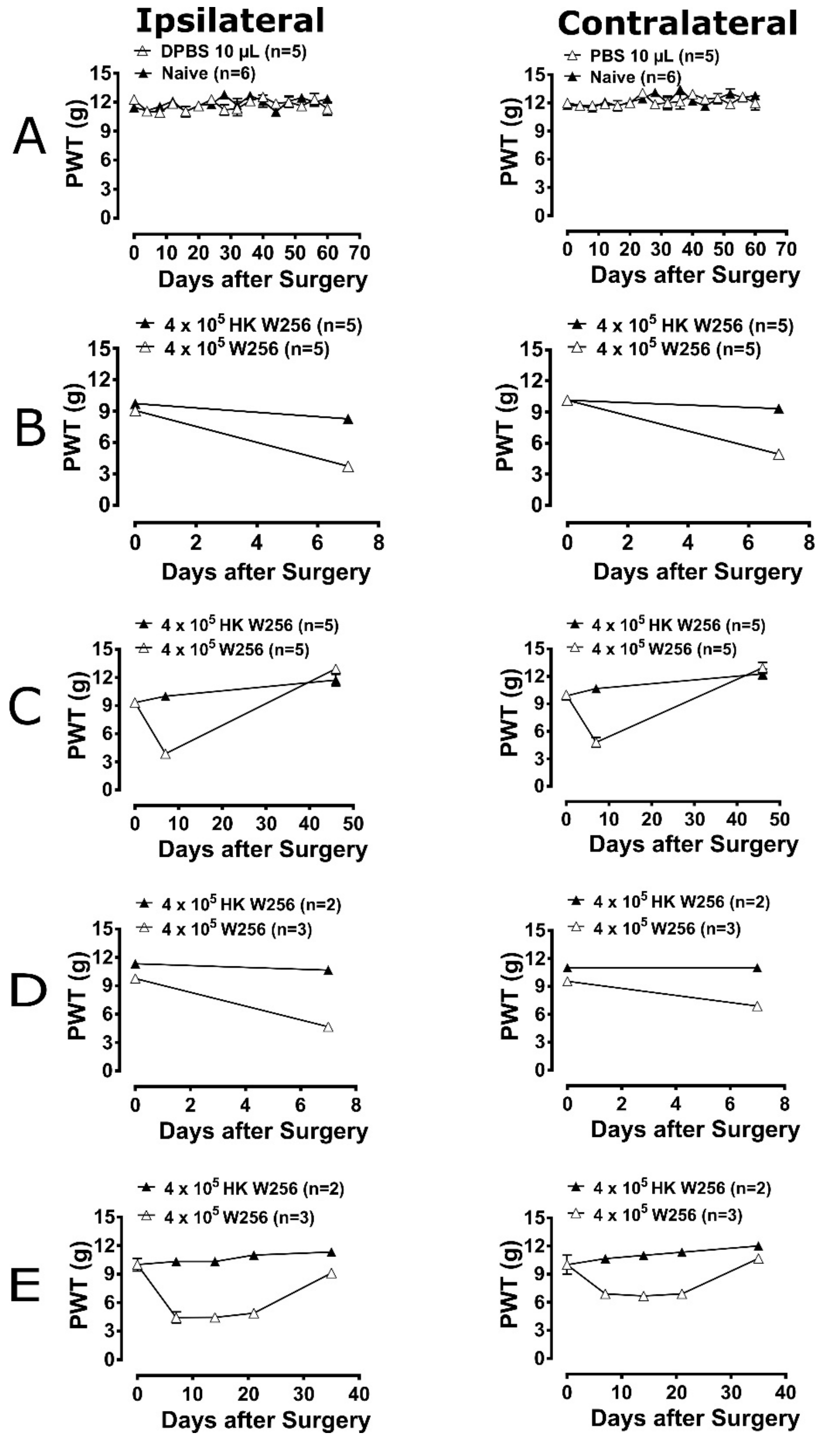

Supplementary Figure 3. Paw withdrawal thresholds (PWTs) of ipsilateral and contralateral hindpaws of rats. Panels in the figure show mean (±SEM) PWTs of rats from (A) experiment 4, (B) experiment 8, (C) experiment 9, (D) experiment 15 and (E) experiment 16. HK, heat-killed; W256, Walker 256.

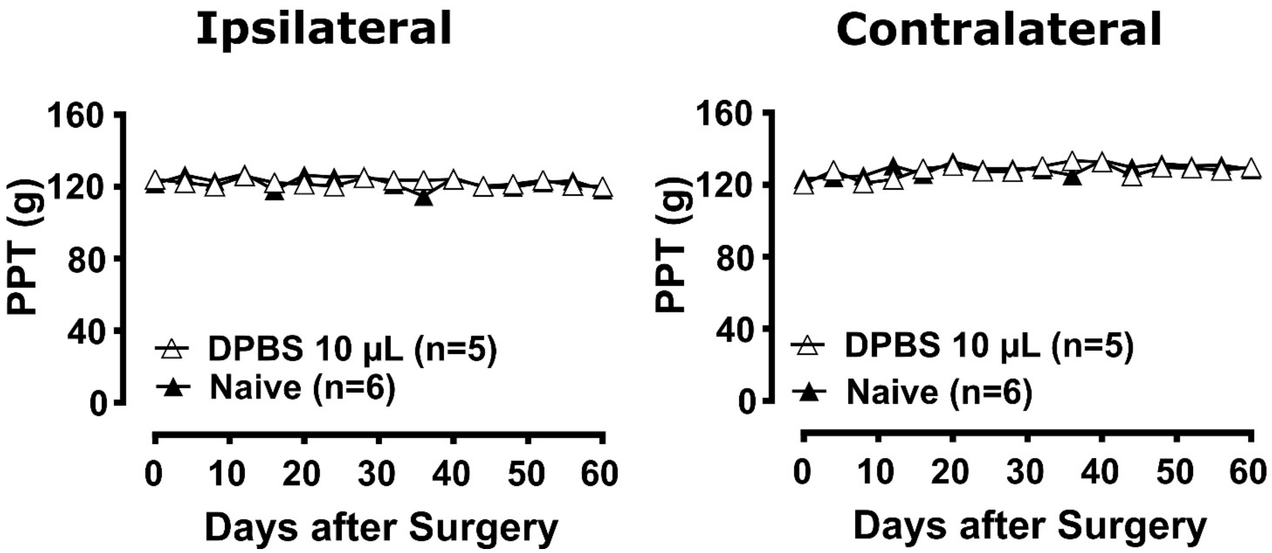

Supplementary Figure 4. Paw pressure thresholds (PPTs) of ipsilateral and contralateral hindpaws of rats from experiment 4.

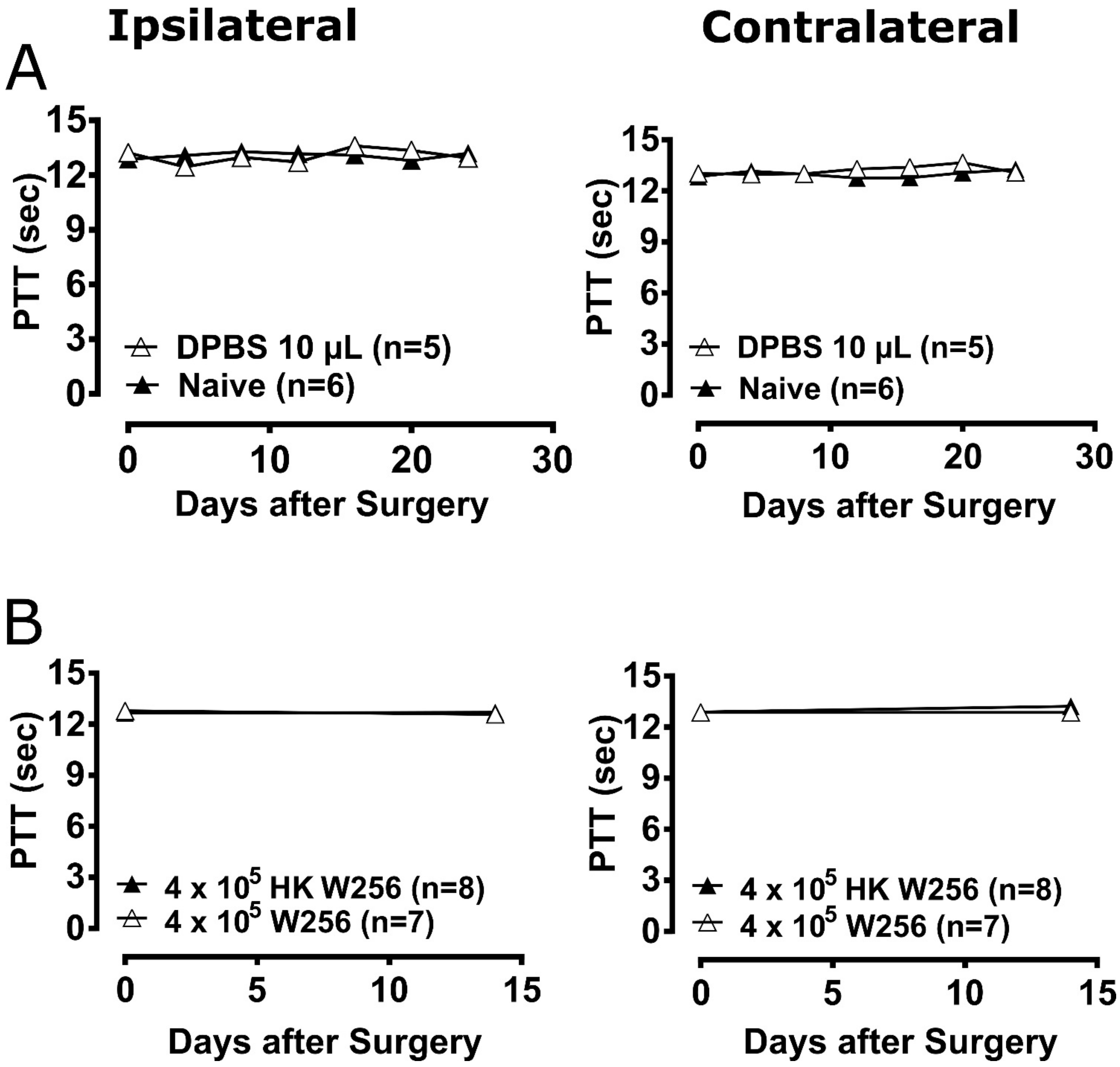

Supplementary Figure 5. Paw thermal thresholds (PTTs) of ipsilateral and contralateral hindpaws of rats. Panels in the figure show mean (±SEM) PTTs of rats from (A) experiment 4 and (B) experiment 5. HK, heat-killed; W256, Walker 256.

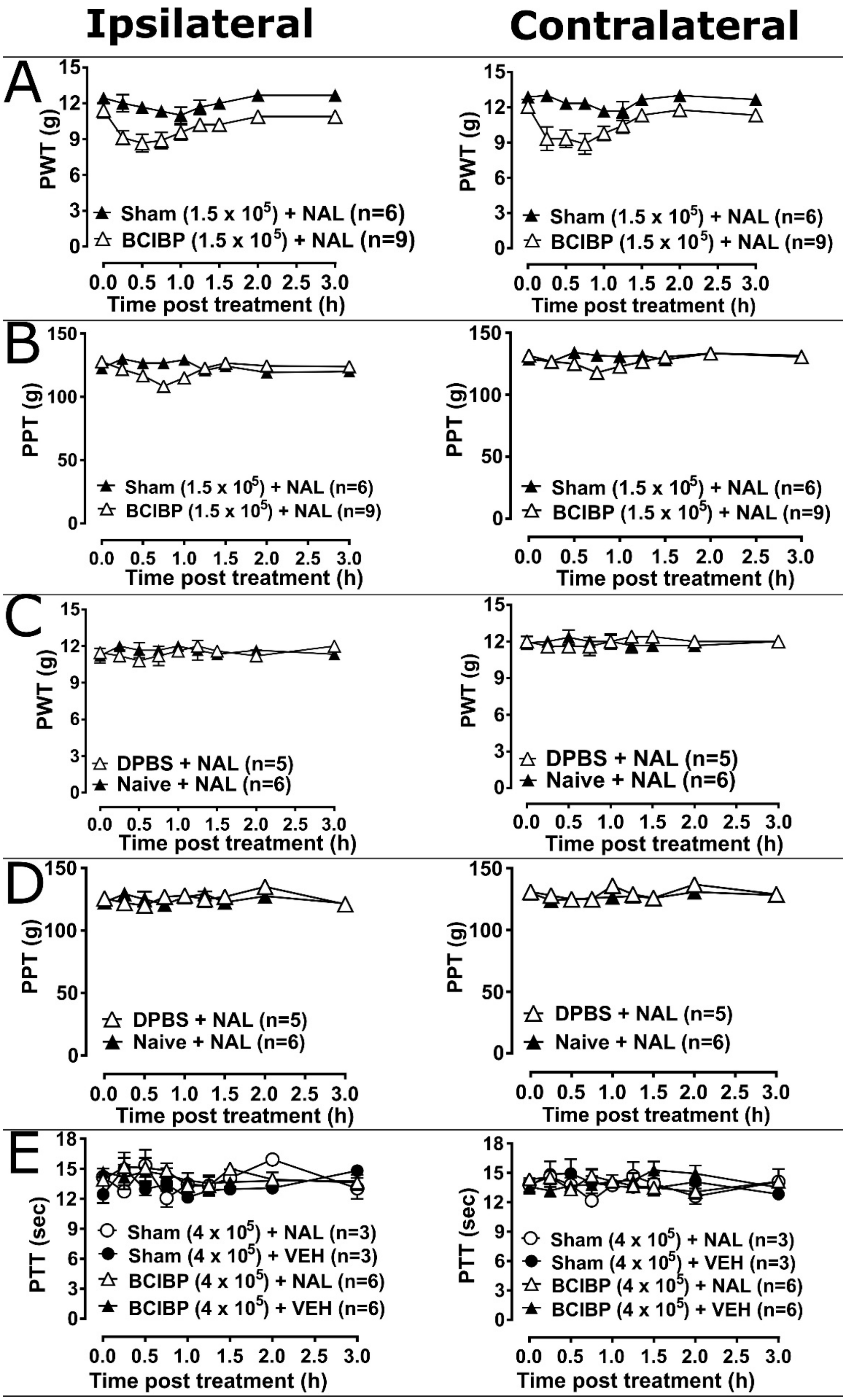
Ipsilateral
Contralateral
A
PWT (g)
Sham (1.5 x 10^5) + NAL (n=6)
BCIBP (1.5 x 10^5) + NAL (n=9)
Time post treatment (h)
B
PPT (g)
Sham (1.5 x 10^5) + NAL (n=6)
BCIBP (1.5 x 10^5) + NAL (n=9)
Time post treatment (h)
C
PWT (g)
DPBS + NAL (n=5)
Naive + NAL (n=6)
Time post treatment (h)
D
PPT (g)
DPBS + NAL (n=5)
Naive + NAL (n=6)
Time post treatment (h)
E
PTT (sec)
Sham (4 x 10^5) + NAL (n=3)
Sham (4 x 10^5) + VEH (n=3)
BCIBP (4 x 10^5) + NAL (n=6)
BCIBP (4 x 10^5) + VEH (n=6)
Time post treatment (h)

Supplementary Figure 6. Effect of naloxone on ipsilateral and contralateral PWT / PPT / PTT values of rats. Panels in the figure show changes in mean (±SEM) (A) PWTs in experiment 3 following naloxone injection between day 81-91 post-ITI, (B) PPTs in experiment 3 following naloxone injection between day 82-92 post-ITI, (C) PWTs in experiment 4 following naloxone injection between day 81-93 post-ITI, (D) PPTs in experiment 4 following naloxone injection between day 82-94 post-ITI and (E) PTTs in experiment 7 following naloxone injection between day 21-24 post-ITI. BCIBP (1.5 x 10^5), group of rats given an ITI of 1.5 x 10^5 W256 cells; BCIBP (4 x 10^5), group of rats given an ITI of 4 x 10^5 W256 cells; DPBS, group of rats given an ITI of 10 µL DPBS; HK, heat-killed; NAL, naloxone (15 mg/kg s.c.); Sham (1.5 x 10^5), group of rats given an ITI of 1.5 x 10^5 HK W256 cells; Sham (4 x 10^5), group of rats given an ITI of 4 x 10^5 HK W256 cells; VEH, vehicle; W256, Walker 256.

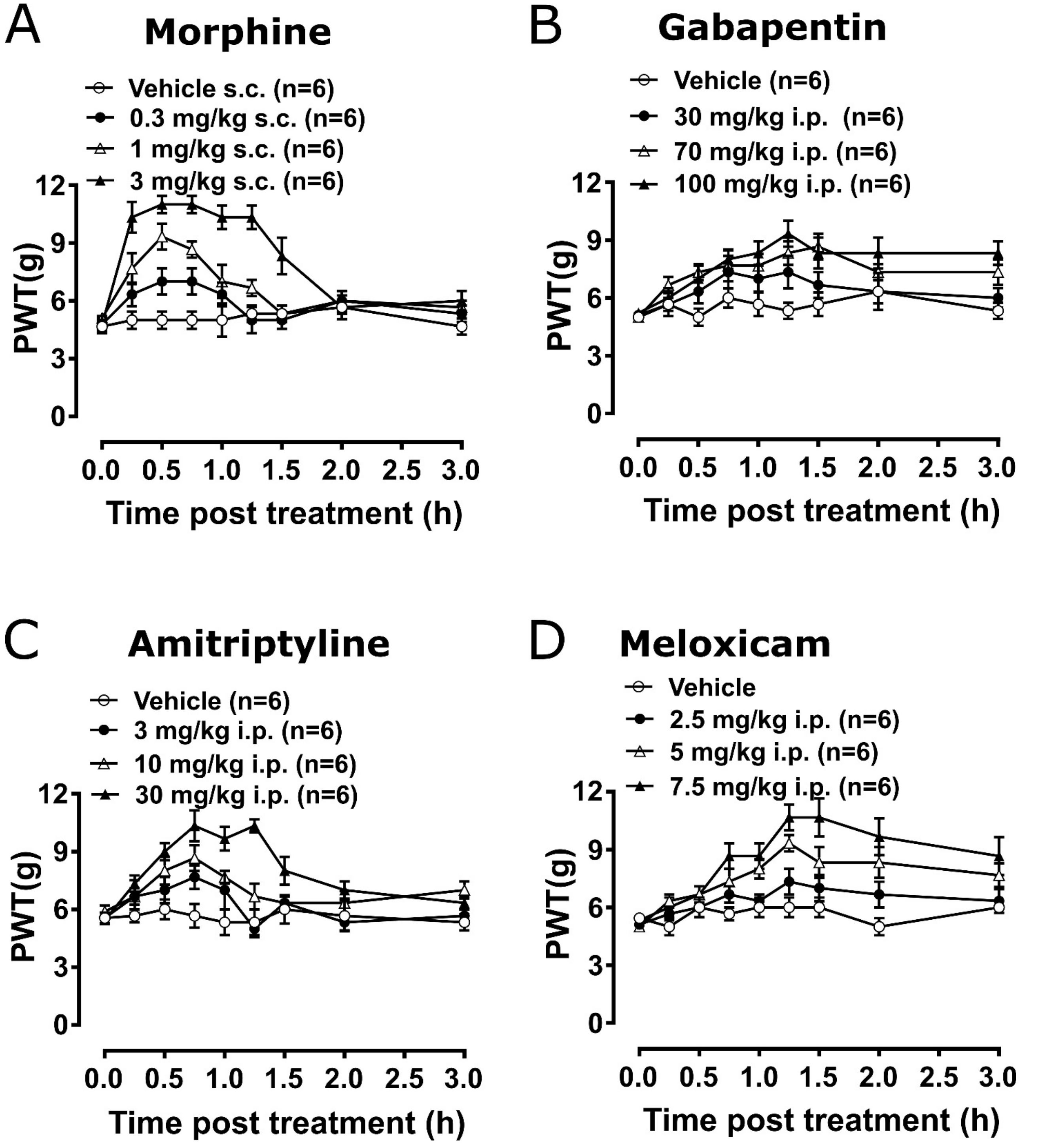

Supplementary Figure 7. Temporal changes in paw withdrawal thresholds (PWTs) of BCIBP rats in contralateral hindpaws following administration of single bolus doses of analgesic and adjuvant drugs. Panels in the figure show temporal changes in mean (±SEM) PWTs versus time curves following injection of (A) morphine, (B) gabapentin, (C) amitriptyline and (D) meloxicam.

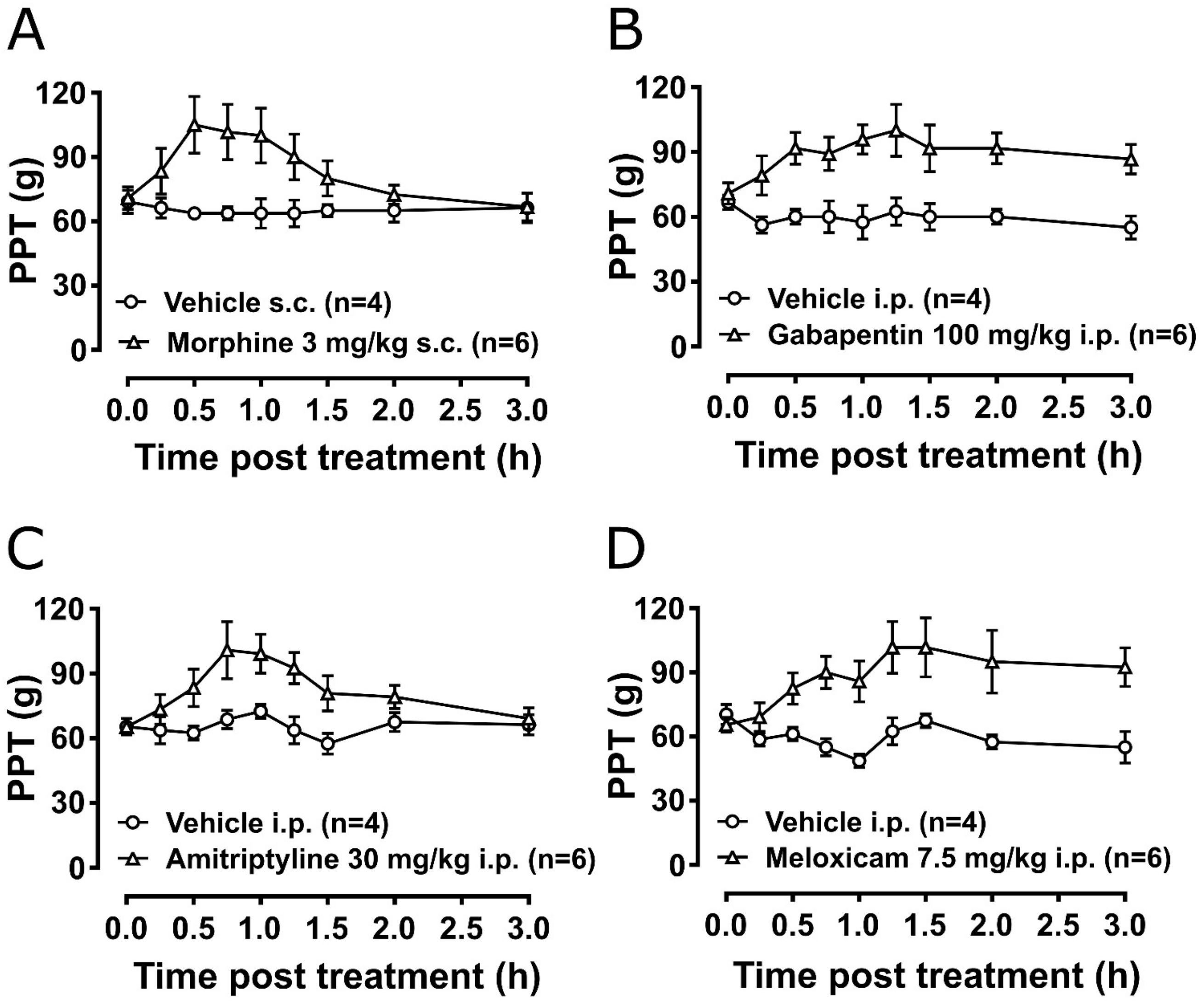

Supplementary Figure 8. Temporal changes in paw pressure thresholds (PPTs) of BCIBP rats in contralateral hindpaws following administration of single bolus doses of analgesic and adjuvant drugs. Panels in the figure show temporal changes in mean (±SEM) PPTs versus time curves following injection of (A) morphine, (B) gabapentin, (C) amitriptyline and (D) meloxicam.

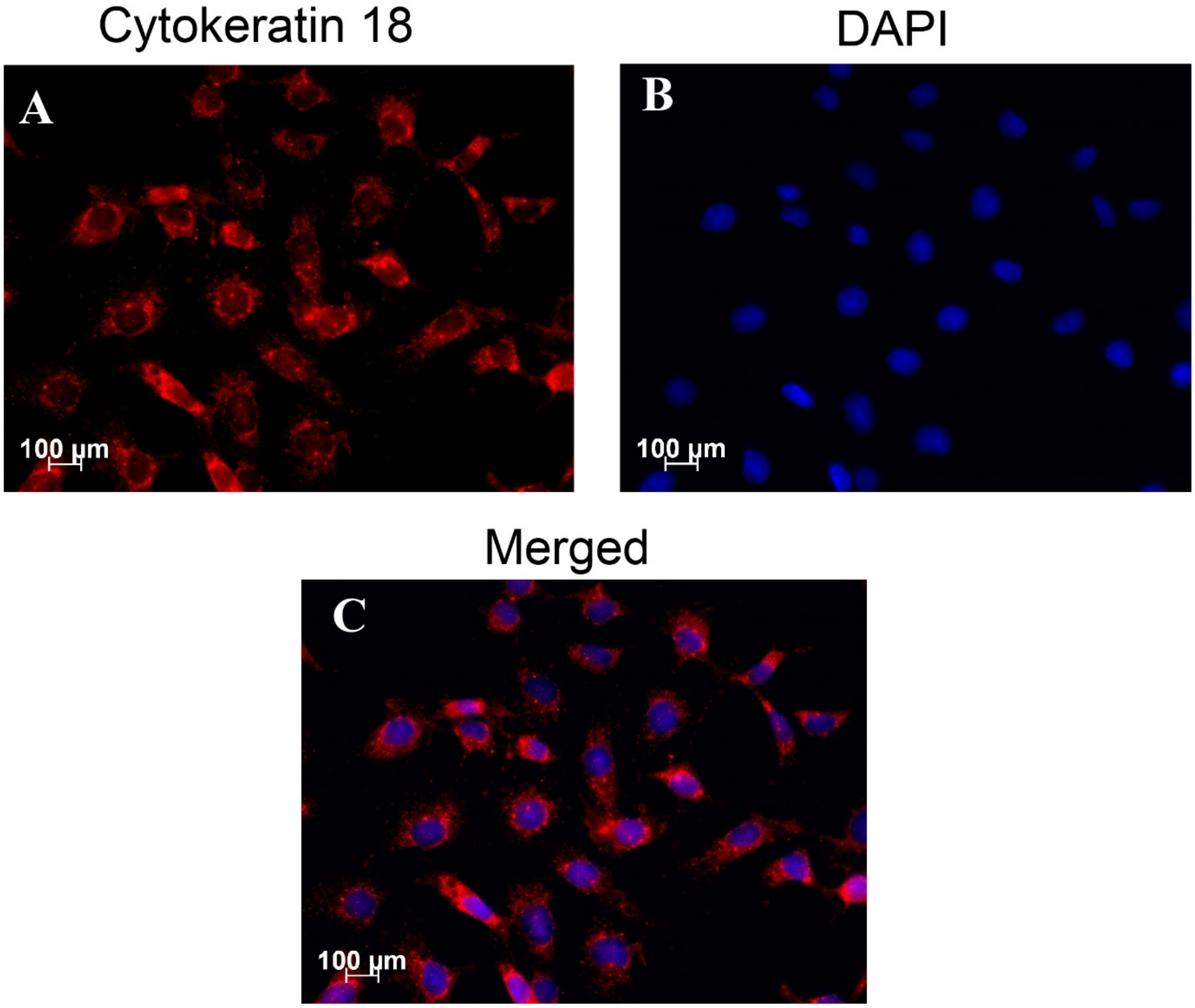

Supplementary Figure 9. Immunocytochemical staining of Walker 256 cell line for Cytokeratin 18 using ab668 (Abcam) antibody. Panels in the figure show (A) cytokeratin 18 (B) DAPI and (C) A and B merged.

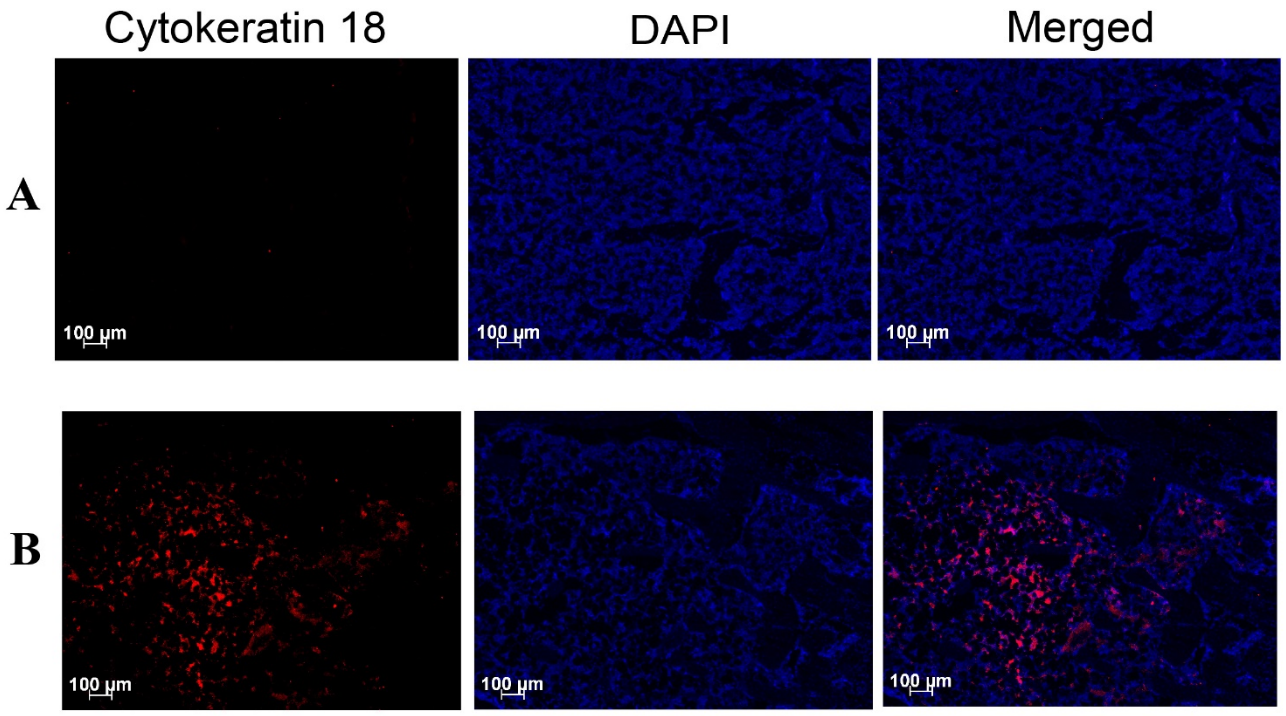

Supplementary Figure 10. Immunohistochemical staining of Cytokeratin 18 in tibial sections of BCIBP rats and corresponding sham rats using ab668 (Abcam) antibody. Panels in the figure show immunofluorescence imaging of tibial sections from (A) sham and (B) BCIBP rats at day 7 post-ITI.
